# RIPK1 autophosphorylation at S161 mediates cell death and inflammation

**DOI:** 10.1084/jem.20250279

**Published:** 2025-09-25

**Authors:** Lioba Koerner, Xiaoming Li, Eveline Silnov, Lucie Laurien, Manolis Pasparakis

**Affiliations:** 1 Institute for Genetics, University of Cologne, Cologne, Germany; 2 Cologne Excellence Cluster for Aging and Aging Associated Diseases (CECAD), University of Cologne, Cologne, Germany; 3 Center for Molecular Medicine (CMMC), Medical Faculty and University Hospital Cologne, University of Cologne, Cologne, Germany

## Abstract

RIPK1 regulates cell death and inflammation and has been implicated in the pathogenesis of inflammatory diseases. RIPK1 autophosphorylation promotes cell death induction; however, the underlying mechanisms and the role of specific autophosphorylation sites remain elusive. Using knock-in mouse models, here we show that S161 autophosphorylation has a critical physiological function in RIPK1-mediated cell death and inflammation. S161N substitution partially suppressed RIPK1-mediated catalytic activity and cell death induction but was sufficient to prevent skin inflammation induced by keratinocyte necroptosis or apoptosis in relevant mouse models. Combined S161N and S166A mutations synergized to prevent RIPK1-mediated cell death more efficiently than the single site mutations, revealing functional redundancy. Moreover, phosphomimetic S161E mutation could overcome the necroptosis-inhibitory effect of S166A mutation, revealing that S161 phosphorylation is sufficient for necroptosis induction. Collectively, a functional interplay of S161 and S166 phosphorylation events regulates RIPK1-dependent cell death and inflammation.

## Teaser

Koerner et al. show that RIPK1 autophosphorylation at S161 is crucial for regulating cell death and inflammation. S161N mutation reduces RIPK1 kinase activity and prevents RIPK1-dependent keratinocyte death and skin inflammation, with combined mutations revealing functional interplay between S161 and S166 phosphorylation.

## Introduction

Receptor-interacting protein kinase 1 (RIPK1) is a critical regulator of cell death and inflammation and is implicated in the pathogenesis of several diseases (Li and Yuan, 2023; Newton, 2020; Varfolomeev and Vucic, 2022). RIPK1 signals downstream of multiple innate immune receptors, including tumor necrosis factor receptor 1 (TNFR1), Toll-like receptor 3 (TLR3), and TLR4, where it induces pro-survival, pro-inflammatory, as well as cell death responses (Li and Yuan, 2023; Newton, 2020; Varfolomeev and Vucic, 2022). RIPK1 acts as a scaffold to induce pro-survival and pro-inflammatory signaling, a function that is critical for mouse development and tissue homeostasis (Dannappel et al., 2014; Dillon et al., 2014; Imai et al., 2024; Kaiser et al., 2014; Kelliher et al., 1998; Lin et al., 2016; Newton et al., 2016b; Rickard et al., 2014b; Takahashi et al., 2014). In contrast, RIPK1 kinase activity induces cell death by activating either caspase-8–dependent apoptosis or RIPK3-mixed lineage kinase like (MLKL)-dependent necroptosis (Kondylis and Pasparakis, 2019; Li and Yuan, 2023; Newton, 2020; Pasparakis and Vandenabeele, 2015; Varfolomeev and Vucic, 2022). The kinase activity of RIPK1 is tightly controlled and several kinases, including inhibitor of NF-κB kinases (IKKs), MAPK 2 (MK2), transforming growth factor β-activating kinase 1 (TAK1), as well as TANK-binding kinase 1 (TBK1) and IKKε, were reported to phosphorylate RIPK1 to suppress its kinase activity and therefore cell death signaling (Dondelinger et al., 2019; Dondelinger et al., 2017; Dondelinger et al., 2015; Eren et al., 2024; Geng et al., 2017; Jaco et al., 2017; Lafont et al., 2018; Menon et al., 2017; Xu et al., 2018). Multiple studies using genetic or pharmacological approaches have identified RIPK1 kinase activity–dependent cell death as a potent trigger of inflammation in different tissues (Berger et al., 2014; Duprez et al., 2011; Eren et al., 2024; Kondylis et al., 2015; Kumari et al., 2021; Polykratis et al., 2019; Schunke et al., 2021; Schwarzer et al., 2020; Vlantis et al., 2016). Furthermore, RIPK1 kinase activity emerged as a driver of ischemic injury (Chen et al., 2018; Naito et al., 2020; Newton et al., 2016a) as well as neurodegenerative diseases such as multiple sclerosis (Ofengeim et al., 2015), amyotrophic lateral sclerosis (Xu et al., 2018), and Alzheimer’s disease (Ofengeim et al., 2017). These studies prompted the development and clinical testing of RIPK1 kinase inhibitors (Clot et al., 2024; Hamilton et al., 2019; Harris et al., 2017; Harris et al., 2019; Hincelin-Mery et al., 2024; Jones et al., 2023; Lickliter et al., 2023; Ludbrook et al., 2024; Patel et al., 2020; Sheridan, 2019; Sun et al., 2024; Vissers et al., 2022; Weisel et al., 2020; Weisel et al., 2021a; Weisel et al., 2021b; Weisel et al., 2017).

The role of RIPK1 kinase activity in inducing cell death and inflammatory pathologies is well established; however, the mechanisms by which RIPK1 catalytic activity induces downstream signaling remain poorly understood. While RIPK1 was initially proposed to phosphorylate other substrates such as DRP1 (Wang et al., 2014), autophosphorylation is currently considered the main and critical function of RIPK1 kinase activity. Autophosphorylation is a common mechanism of autoactivation in eukaryotic protein kinases, which leads to the stabilization of a specific protein conformation, allowing for efficient catalysis (Beenstock et al., 2016). The currently prevailing model is that autophosphorylation induces conformational changes in RIPK1 that allow its association with cell death effectors including Fas-associated with death domain (FADD) and receptor-interacting protein kinase 3 (RIPK3), facilitating the formation of cell death–inducing signaling complexes causing apoptosis or necroptosis (Wegner et al., 2017). In support of this hypothesis, RIPK1 autophosphorylation was shown to promote the ordered assembly of RIPK1 homo-oligomers as a prerequisite for RIPK3 oligomerization and necrosome formation (Chen et al., 2022). RIPK1 was reported to autophosphorylate on multiple sites in the kinase domain, including S14/15, S20, S161, and S166 in humans (Degterev et al., 2008) and S14/15, S161, S166, and T169 in mice (Berger et al., 2014; Dondelinger et al., 2019; Laurien et al., 2020; Ofengeim et al., 2015). Of note, S161, S166, and T169 are located in the RIPK1 activation loop, a functionally important element for kinase activation. Several studies have used in vitro cellular systems to address the importance of different autophosphorylation sites for RIPK1-mediated cell death. A phosphomimetic S161E mutation was reported to overcome the kinase-inactivating K45A mutation in induction of cell death, suggesting that S161 is the crucial site that transmits the lethal stimulus (Zhang et al., 2017). Furthermore, an S161N mutation disrupted the formation of ordered RIPK1 oligomers required for necrosome formation (Chen et al., 2022). However, other studies assigned less functional importance to this site (Degterev et al., 2008), the function of which has not been validated in vivo. On the other hand, autophosphorylation at S166 is used as a standard biomarker for RIPK1 activation facilitated by the development of specific antibodies (Berger et al., 2014; Dondelinger et al., 2017; Newton et al., 2016a; Patel et al., 2020). We have previously reported that RIPK1 autophosphorylation at S166 is critical for RIPK1 kinase–dependent cell death and inflammation (Laurien et al., 2020). However, S166A mutation was not sufficient to fully prevent RIPK1-mediated necroptosis and apoptosis in vitro, suggesting that other autophosphorylation events also contribute to RIPK1 activation (Laurien et al., 2020).

Here we addressed the physiological role of autophosphorylation at S161 alone or in combination with S166 in the regulation of RIPK1-mediated cell death and inflammation using knock-in mice expressing RIPK1 with respective mutations. Our results revealed that S161 and S166 exhibit partially redundant functions in regulating RIPK1 kinase activation and cell death induction. Furthermore, we found that phosphomimetic S161E mutation could partially overcome the protective effect of S166A and specifically sensitized cells to necroptosis. Thus, autophosphorylation events at S161 and S166 cooperate to regulate RIPK1-mediated cell death and inflammation in vitro and in vivo.

## Results

### S161N, but not S161A, mutation prevents RIPK1 kinase activity–dependent necroptosis and inflammation

**Figure S1.**
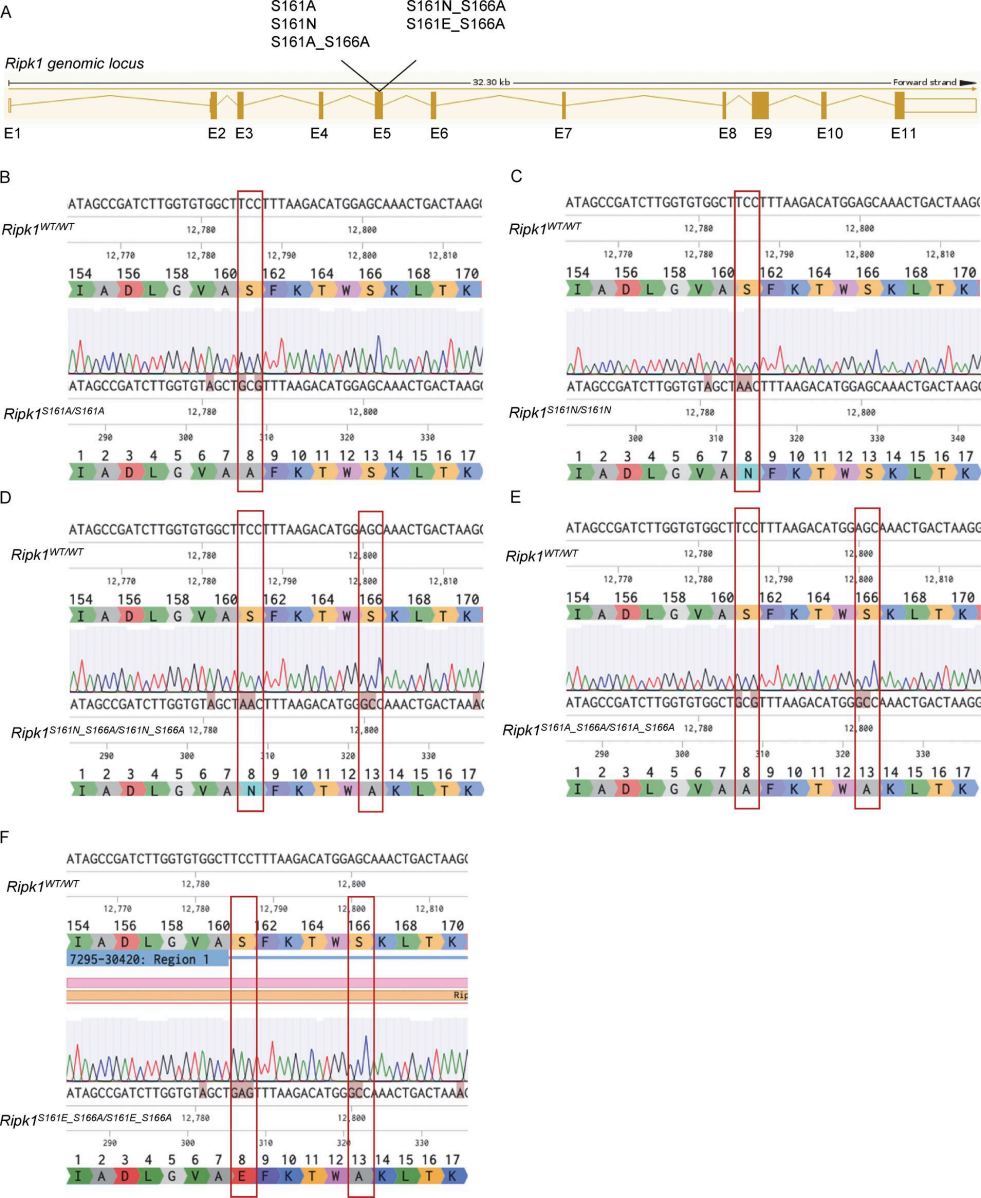
**Generation of RIPK1 autophosphorylation site mutant mice. (A)** The residues S161 and S166 in exon 5 of the *Ripk1* gene were altered from serine to alanine (S161A), from serine to asparagine (S161N), and from serine to glutamic acid (S161E) using CRISPR/Cas9 technology. Oocytes fertilized with sperm of *Ripk1*^*S166A/S166A*^ males or WT mice were electroporated with knock-in oligos and Cas9 protein for the respective mouse lines. The sequences for all knock-in oligos can be found in the Materials and methods section. **(B)** DNA sequence alignment (5′-3′) of the region surrounding the mutation site in WT and *Ripk1*^*S161A/S161A*^ mice. Amino acids by triplet code are shown. The mutation is marked in red. **(C)** DNA sequence alignment (5′-3′) of the region surrounding the mutation site in WT and *Ripk1*^*S161N/S161N*^ mice. Amino acids by triplet code are shown. The mutation is marked in red. **(D)** DNA sequence alignment (5′-3′) of the region surrounding the mutation site in WT and *Ripk1*^*S161N_S166A/S161N_S166A*^ mice. Amino acids by triplet code are shown. The mutations are marked in red. **(E)** DNA sequence alignment (5′-3′) of the region surrounding the mutation site in WT and *Ripk1*^*S161A_S166A/S161A_S166A*^ mice. Amino acids by triplet code are shown. The mutations are marked in red. **(F)** DNA sequence alignment (5′-3′) of the region surrounding the mutation site in WT and *Ripk1*^*S161E_S166A/S161E_S166A*^ mice. Amino acids by triplet code are shown. The mutations are marked in red.

**Figure 1.**
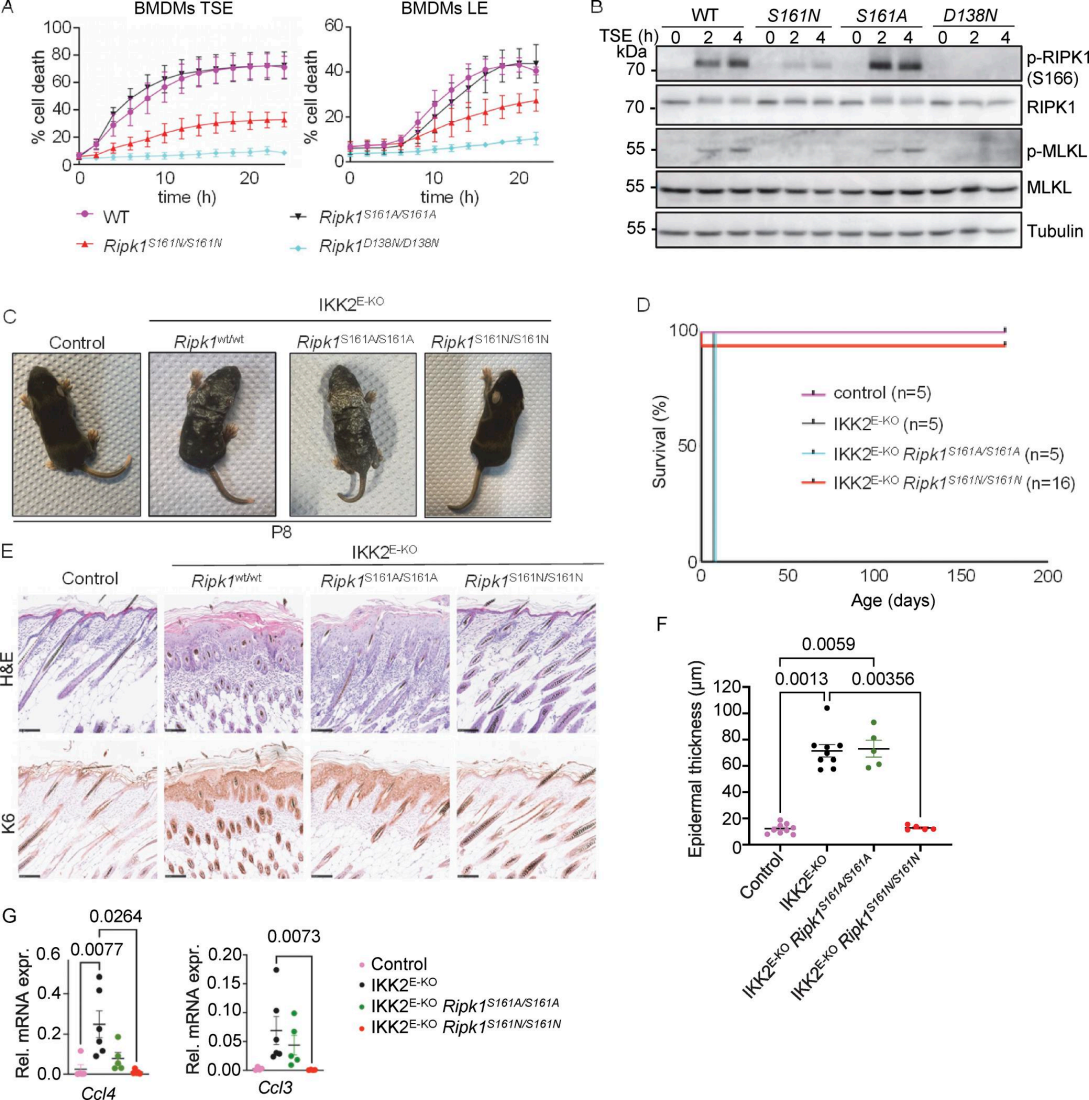
**Autophosphorylation at S161 drives RIPK1 kinase activity–dependent necroptosis. (A)** Graphs depicting quantification of cell death in BMDMs from mice of the indicated genotypes treated with combinations of Emricasan (E, 5 µM), the SMAC mimetic compound birinapant (S, 1 µM), TNF (T, 20 ng/ml), and LPS (L, 100 ng/ml). Graphs show mean ± SEM from at least four independent experiments. **(B)** Immunoblots of BMDMs from mice of the indicated genotypes stimulated with TSE for 0, 2, or 4 h. Representative of three independent experiments. **(C)** Representative photographs of control (*n* = 8), IKK2^E-KO^ (*n* = 7), IKK2^E-KO^*Ripk1*^*S161A/S161A*^ (*n* = 5), and IKK2^E-KO^*Ripk1*^*S161N/S161N*^ (*n* = 5) mice. **(D)** Kaplan–Meier survival curve of mice with the indicated genotypes. **(E)** Representative images of sections from back skin of mice with the indicated genotypes stained with H&E or immunostained for K6 (scale bars = 100 µm; control *n* = 7, IKK2^E-KO^*n* = 7, IKK2^E-KO^*Ripk1*^*S161A/S161A*^*n* = 4, and IKK2^E-KO^*Ripk1*^*S161N/S161N*^*n* = 4). **(F)** Graph depicting epidermal thickness of mice with the indicated genotypes. Each dot represents one mouse. Mean ± SEM are shown. Statistical significance was determined using Kruskal–Wallis test. **(G)** Graphs depicting relative mRNA expression of the indicated cytokines in RNA from whole-skin tissue of mice of the indicated genotypes measured by qRT-PCR. Each dot represents one mouse. Mean ± SEM are shown. Statistical significance was determined using Kruskal–Wallis test. Control mice include *Ikk2*^FL/FL^*K14Cre*^WT/WT^ or *Ikk2*^FL/WT^*K14Cre*^Tg/WT^ littermates with WT or mutant *Ripk1* alleles. Source data are available for this figure: SourceData F1.

Previous studies based on cellular overexpression systems suggested a functional role of S161 phosphorylation in RIPK1-mediated cell death (Zhang et al., 2017). To study the role of autophosphorylation at S161 in a physiological setting, we employed CRISPR-Cas9–mediated gene targeting to generate knock-in mice expressing RIPK1 with a non-phosphorylatable amino acid replacement at this site (Fig. S1, A–C). Of note, serine at position 161 was suggested to maintain the closed conformation of the RIPK1 activation loop in the inactive kinase via hydrogen bond formation with D156 (Zhang et al., 2017). In previous studies, S161 was replaced by either alanine (S161A) or asparagine (S161N), which differ in their capability to form hydrogen bonds. Specifically, substitution of S161 with alanine is predicted to weaken the hydrogen bond with D156, thus favoring an open conformation of RIPK1, while replacement of S161 with asparagine should retain the hydrogen bonds with D156, maintaining the closed conformation. Thus, to comprehensively study the role of this site, we used both approaches side by side and generated *Ripk1*^S161A/S161A^ and *Ripk1*^S161N/S161N^ mice (Fig. S1, A–C). These mice were born at the expected Mendelian ratios and reached adulthood without exhibiting apparent abnormalities (data not shown). To address the role of S161 autophosphorylation in RIPK1 kinase activity–dependent necroptosis, we assessed cell death in bone marrow–derived macrophages (BMDMs) from *Ripk1*^S161A/S161A^ and *Ripk1*^S161N/S161N^ mice after stimulation with TNF in combination with the SMAC mimetic compound birinapant and the caspase inhibitor Emricasan (TSE). As shown in Fig. 1 A, BMDMs from *Ripk1*^S161A/S161A^ mice showed similar cell death kinetics compared to WT BMDMs, demonstrating that the S161A substitution did not prevent RIPK1 kinase–dependent necroptosis. In contrast, *Ripk1*^S161N/S161N^ BMDMs were protected from TSE-induced cell death, although this protection was not as complete as that observed in *Ripk1*^D138N/D138N^ BMDMs, which express kinase-inactive RIPK1 (Polykratis et al., 2014) (Fig. 1 A). Assessment of cell death following stimulation with LPS in combination with Emricasan showed comparable results, with *Ripk1*^S161A/S161A^ BMDMs displaying cell death kinetics similar to WT cells, whereas *Ripk1*^S161N/S161N^ BMDMs exhibited partial protection (Fig. 1 A). In line with the cell death assay results, the S161N mutation suppressed TSE-induced MLKL phosphorylation, while the S161A mutation did not (Fig. 1 B). To assess how the S161 mutations affected RIPK1 autophosphorylation at S166, we immunoblotted with antibodies recognizing specifically phosphorylated S166. RIPK1 phosphorylation at S166 was not suppressed in TSE-stimulated *Ripk1*^S161A/S161A^ BMDMs, while it was strongly, but not completely, inhibited in *Ripk1*^S161N/S161N^ cells (Fig. 1 B). Together, these findings showed that S161N, but not S161A, substitution partially inhibited RIPK1 autophosphorylation on S166 and RIPK1 kinase–dependent necroptosis.

To assess how the S161A and S161N substitutions affected RIPK1 kinase–dependent necroptosis and inflammation in vivo, we employed mice that lack IKK2/IKKβ specifically in epidermal keratinocytes (IKK2^E-KO^) and develop severe skin inflammation due to TNFR1-RIPK1–mediated keratinocyte necroptosis (Kumari et al., 2013; Kumari et al., 2021; Pasparakis et al., 2002). IKK2^E-KO^ mice develop skin lesions during the first week of life that progress in severity, reaching the ethical endpoint by postnatal day 8 (P8), when they display macroscopically scaly rigid skin (Fig. 1, C and D). Our previous genetic studies revealed that skin inflammation in IKK2^E-KO^ mice is mainly driven by RIPK3-MLKL–mediated keratinocyte necroptosis and, to a lesser extent, by FADD–caspase-8–dependent cell death (Kumari et al., 2021). IKK2^E-KO^*Ripk1*^S161A/S161A^ mice developed skin lesions with similar kinetics compared to IKK2^E-KO^ pups (Fig. 1, C and D). In contrast, IKK2^E-KO^*Ripk1*^S161N/S161N^ mice did not show skin alterations at P8 and remained free of skin lesions until at least 25 wk of age (Fig. 1, C and D). Histological analysis revealed epidermal thickening with increased cellularity in the dermis and upregulation of keratin 6 (K6) expression in the skin of IKK2^E-KO^ and IKK2^E-KO^*Ripk1*^S161A/S161A^ mice at P8, whereas IKK2^E-KO^*Ripk1*^S161N/S161N^ mice showed normal skin histology at this stage (Fig. 1, E and F). Moreover, IKK2^E-KO^ and IKK2^E-KO^*Ripk1*^S161A/S161A^ mice showed upregulation of *Ccl3* and *Ccl4* in the skin, which was suppressed in IKK2^E-KO^*Ripk1*^S161N/S161N^ mice (Fig. 1 G). Therefore, consistent with our cell culture experiments, S161N, but not S161A substitution prevented RIPK1 kinase activity–dependent keratinocyte necroptosis and skin inflammation in vivo in IKK2^E-KO^ mice. These findings revealed a critical role of S161 in the regulation of RIPK1-mediated necroptosis. Abolishing phosphorylation on this site by S161N substitution strongly suppressed necroptosis in vivo and in vitro. Our finding that S161A substitution did not inhibit RIPK1-mediated necroptosis is in agreement with the previously suggested role of S161 in maintaining the closed conformation of RIPK1 by forming hydrogen bonds with D156, which are weakened by S161A substitution, resulting in a more open RIPK1 conformation even in the absence of phosphorylation on this site (Zhang et al., 2017).

### S161N, but not S161A, mutation prevents RIPK1 kinase activity–dependent apoptosis and inflammation

**Figure S2.**
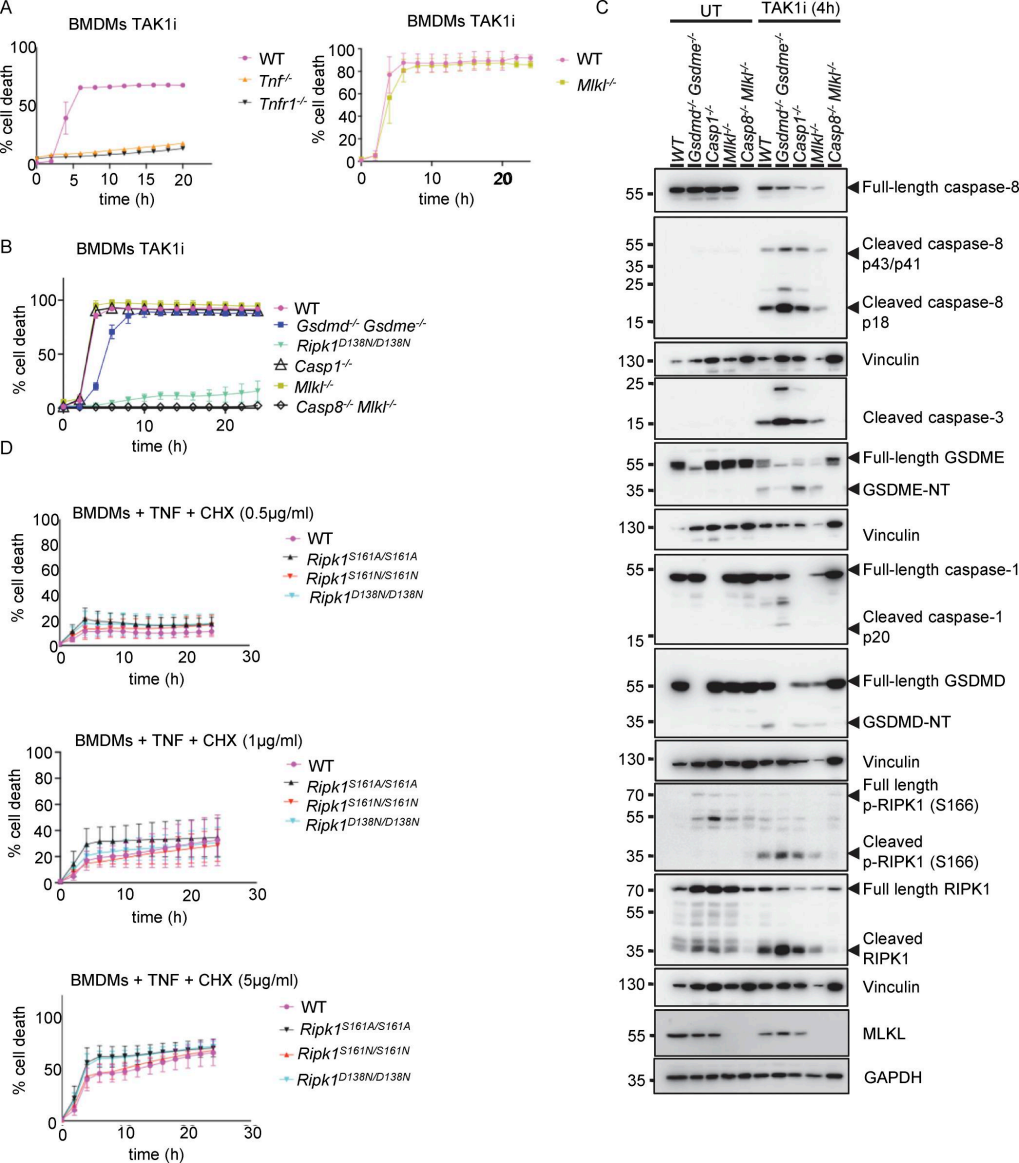
**TAK1 inhibitor triggers caspase-8–dependent apoptosis induced by autocrine TNF-TNFR1 signaling in BMDMs. (A and B)** Graphs depicting quantification of cell death in BMDMs from mice of the indicated genotypes treated with TAK1 inhibitor (TAK1i, 0.25 µM). Graphs show the mean ± SEM of at least two independent experiments. **(C)** Immunoblots of BMDMs from mice of the indicated genotypes either untreated (UT) or treated with TAK1i (0.25 µM) for 4 h. **(D)** Graph depicting quantification of cell death in BMDMs from mice of the indicated genotypes treated with a combination of TNF (T, 20 ng/ml) and the indicated amounts of CHX. Graphs show the mean ± SEM of four independent experiments. Source data are available for this figure: SourceData FS2.

**Figure S3.**
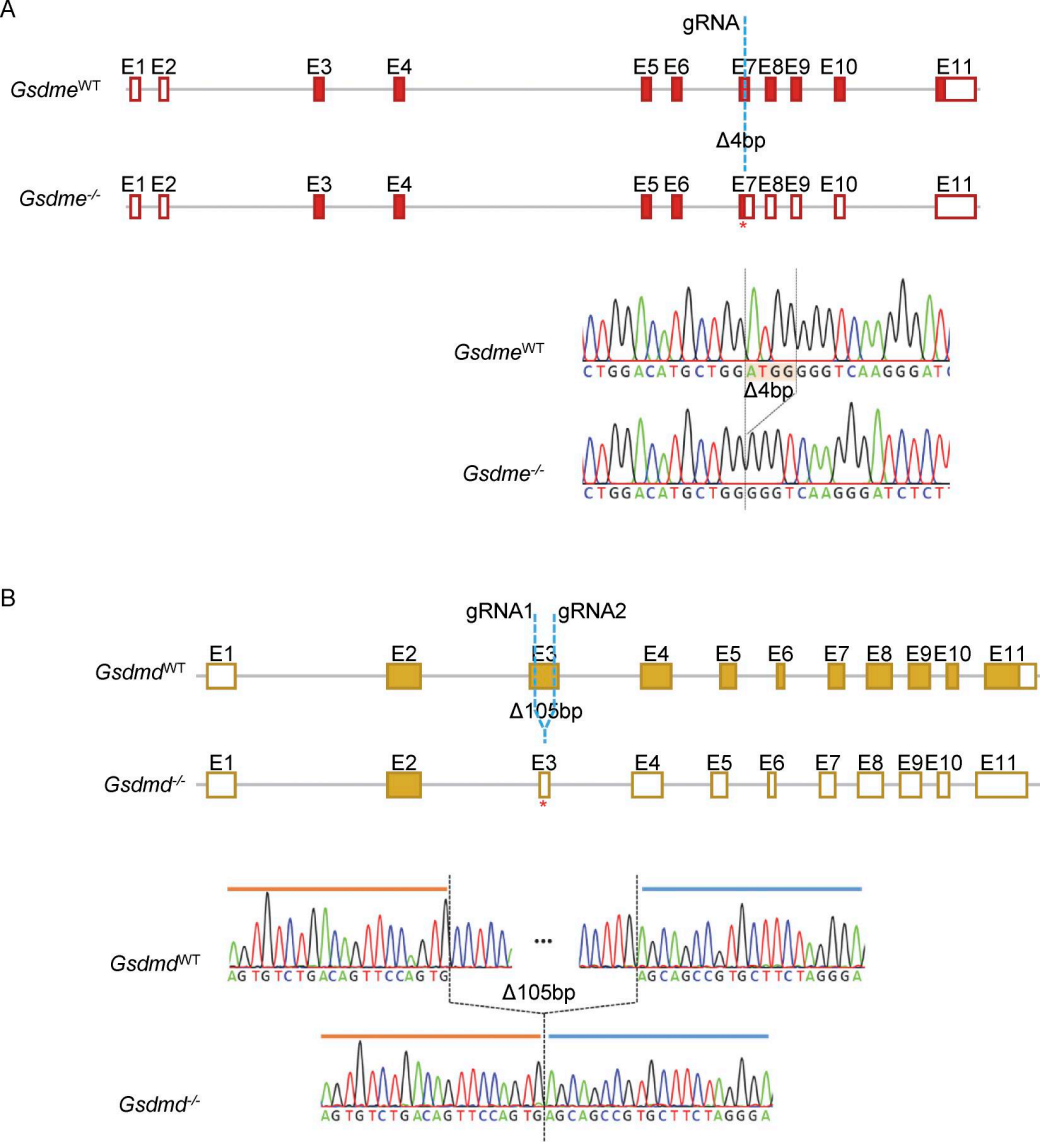
**Generation of *Gsdme*** ^***−/−***^ **and *Gsdmd*** ^***−/−***^ **mice. (A and B)** Schematics depicting the generation of mice that are deficient for GSDME (A) or GSDMD (B) using CRISPR-Cas9–mediated gene targeting in C57BL/6N zygotes, as indicated. To generate *Gsdme*^*−/−*^ mice, exon 7 was targeted, resulting in a 4-bp deletion and therefore a frameshift and a premature stop codon indicated with a star (*). To generate *Gsdmd*^*−/−*^ mice, exon 3 was targeted by two gRNAs, resulting in a 105-bp deletion and a frameshift in exon 3. The new premature stop codon is indicated with a star (*). The resulting deletions were confirmed by Sanger sequencing.

**Figure 2.**
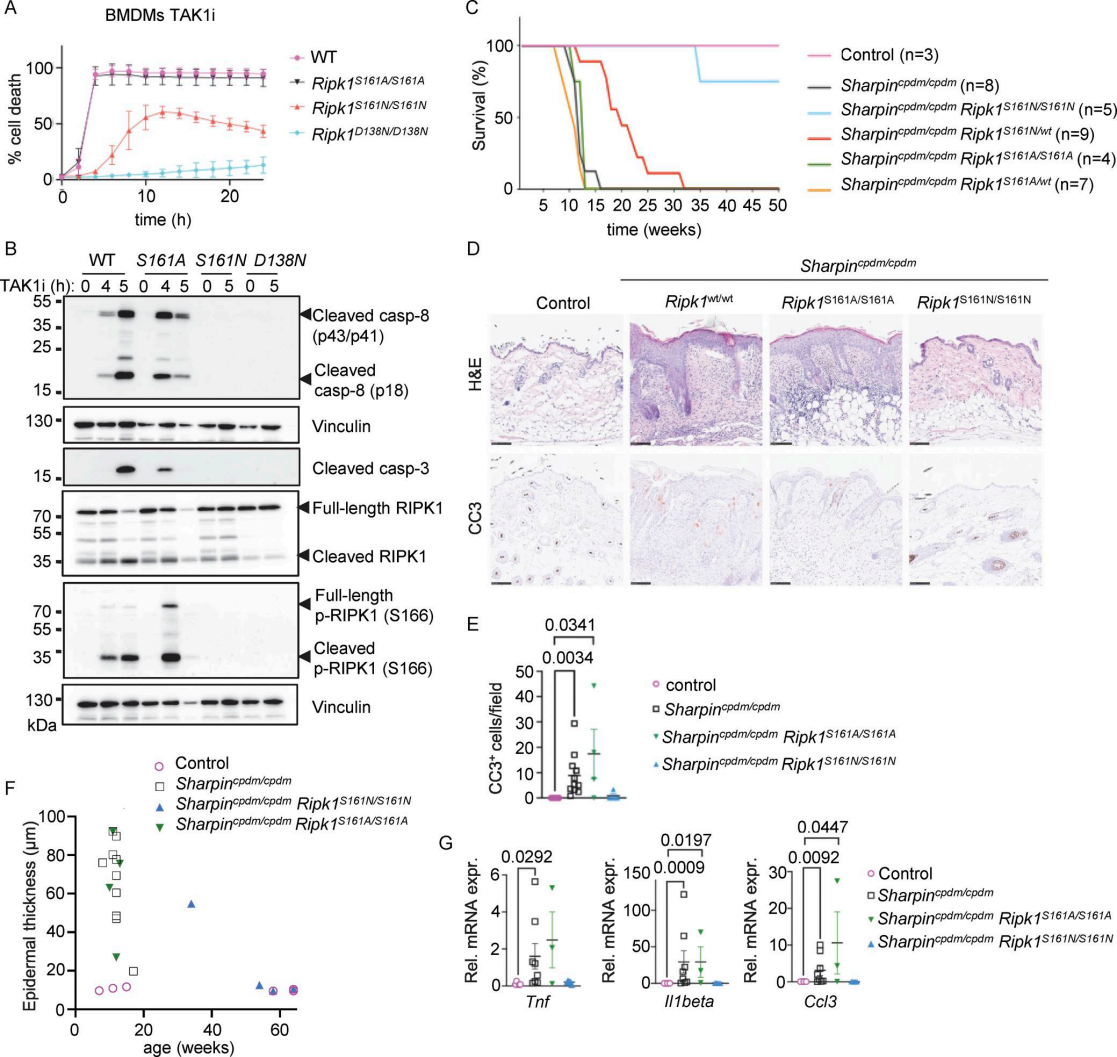
**Autophosphorylation at S161 drives RIPK1 kinase activity–dependent apoptosis. (A)** Graph depicting quantification of cell death in BMDMs from mice of the indicated genotypes treated with TAK1 inhibitor (TAK1i, 0.25 µM). Graph shows mean ± SEM of three independent experiments. **(B)** Immunoblots of BMDMs from mice of the indicated genotypes stimulated with TAK1i for 0, 4, and 5 h. Representative of two independent experiments. **(C)** Kaplan–Meier survival curve of mice with the indicated genotypes. **(D)** Representative images of sections from back skin of mice with the indicated genotypes stained with H&E or immunostained for CC3 (scale bars = 100 µm; control *n* = 5, *Sharpin*^*cpdm/cpdm*^*n* = 10, *Sharpin*^*cpdm/cpdm*^*Ripk1*^*S161A/S161A*^*n* = 4, *Sharpin*^*cpdm/cpdm*^*Ripk1*^*S161N/S161N*^*n* = 4). **(E)** Graph showing the average number of CC3^+^ cells per optical field in mice with the indicated genotypes. Mean ± SEM are shown. Each dot represents one mouse. Statistical significance was determined using Kruskal–Wallis test. **(F)** Graph depicting measurement of epidermal thickness of mice with the indicated genotypes. Each dot represents one mouse. **(G)** Graphs depicting relative mRNA expression of the indicated cytokines in RNA from whole-skin tissue of mice of the indicated genotypes measured by qRT-PCR. Each dot represents one mouse. Mean ± SEM are shown. Statistical significance was determined using Kruskal–Wallis test. Control mice include *Sharpin*^cpdm/WT^ or *Sharpin*^WT/WT^ littermates with WT or mutant *Ripk1* alleles. Source data are available for this figure: SourceData F2.

To assess how S161A and S161N substitutions affected RIPK1 kinase activity–dependent apoptosis, we treated BMDMs with the TAK1 inhibitor 5Z-7 oxozeaenol (TAK1i) (Wu et al., 2013). Treatment with TAK1i induced cell death in BMDMs that was dependent on endogenous TNF and TNFR1 signaling and on caspase-8, but occurred independently of MLKL and caspase-1 and was only slightly delayed in cells lacking gasdermin D (GSDMD) and gasdermin E (GSDME), demonstrating that BMDMs undergo apoptosis under these conditions (Figs. S2 and S3). In response to TAK1i treatment, *Ripk1*^S161A/S161A^ BMDMs underwent rapid cell death, whereas *Ripk1*^D138N/D138N^ cells were protected, showing that the S161A mutation did not hinder RIPK1 kinase–dependent apoptosis (Fig. 2 A). Under the same conditions, *Ripk1*^S161N/S161N^ BMDMs showed considerably slower cell death kinetics and were partially protected from TAK1i-induced cell death compared with WT cells (Fig. 2 A). However, stimulation with TNF in the presence of different concentrations of cycloheximide (CHX), which triggers RIPK1-independent apoptosis, induced similar cell death responses in WT, *Ripk1*^S161A/S161A^, *Ripk1*^S161N/S161N^, and *Ripk1*^D138N/D138N^ BMDMs (Fig. S2 D). In line with the cell death assays, we detected caspase-8 and caspase-3 cleavage in WT and in *Ripk1*^S161A/S161A^ BMDMs 4 and 5 h after treatment with TAK1i, whereas *Ripk1*^S161N/S161N^ BMDMs did not show caspase cleavage at this time point (Fig. 2 B). Therefore, S161N, but not S161A, substitution could partially suppress RIPK1 kinase–dependent apoptosis. Moreover, we detected strong phosphorylation of RIPK1 at S166 in both WT and *Ripk1*^S161A/S161A^ BMDMs, but not in *Ripk1*^S161N/S161N^ cells (Fig. 2 B). Interestingly, S166 phosphorylation was mostly detected at a molecular weight corresponding to the N-terminal cleaved fragment of RIPK1 (Fig. 2 B), suggesting that phosphorylated RIPK1 is rapidly cleaved by caspase-8 in TAK1i-treated BMDMs.

To assess how S161 mutations affect RIPK1 kinase activity–dependent apoptosis and inflammation in vivo, we employed *Sharpin*^cpdm/cpdm^ mice that develop inflammatory skin lesions due to TNFR1-RIPK1-FADD-caspase-8–dependent keratinocyte apoptosis (Berger et al., 2014; Kumari et al., 2014; Laurien et al., 2020; Rickard et al., 2014a). Homozygous or heterozygous S161A mutation did neither prevent nor delay skin inflammation in *Sharpin*^cpdm/cpdm^*Ripk1*^S161A/S161A^ and in *Sharpin*^cpdm/cpdm^*Ripk1*^S161A/wt^ mice, as these animals developed skin lesions with similar kinetics compared to *Sharpin*^cpdm/cpdm^ mice and reached the ethical endpoint between 10–15 wk of age (Fig. 2 C). In contrast, homozygous S161N mutation strongly prevented skin lesions with three out of four animals remaining lesion-free up to the age of 50 wk, while heterozygous S161N mutation also delayed the development of skin lesions to over 20 wk for around 50% of the mice (Fig. 2 C). Of note, the effect of the S161N mutation was comparable with the strong protection observed previously in *Sharpin*^cpdm/cpdm^*Ripk1*^S166A/S166A^ mice (Laurien et al., 2020). Histopathological analysis of skin sections from 10 to 14-wk-old mice revealed epidermal thickening and increased numbers of cleaved caspase-3 positive (CC3^+^) cells in *Sharpin*^cpdm/cpdm^ mice, which were not inhibited by the S161A mutation in *Sharpin*^cpdm/cpdm^*Ripk1*^S161A/S161A^ mice (Fig. 2, D–F), consistent with the macroscopic assessment. In contrast, three out of four *Sharpin*^cpdm/cpdm^*Ripk1*^S161N/S161N^ mice showed normal skin histology and absence of CC3^+^ cells similarly to WT control animals even at 50 wk of age (Fig. 2 F and data not shown). Furthermore, increased mRNA expression of *Tnf*, *Il1b*, and *Ccl3* was detected in the skin of *Sharpin*^cpdm/cpdm^ and *Sharpin*^cpdm/cpdm^*Ripk1*^S161A/S161A^ animals, which was suppressed in *Sharpin*^cpdm/cpdm^*Ripk1*^S161N/S161N^ mice (Fig. 2 G). Therefore, S161N, but not S161A, substitution strongly prevented RIPK1 kinase–dependent keratinocyte apoptosis and skin inflammation in *Sharpin*^cpdm/cpdm^ mice. Taken together, these results revealed that phosphorylation at S161 is critical to license RIPK1-mediated apoptosis and further supported that S161A substitution promotes an open conformation, negating the need for S161 phosphorylation for RIPK1 activation.

### Autophosphorylation at S161 and S166 cooperate to induce RIPK1 kinase activity–dependent cell death in a partially redundant manner

**Figure 3.**
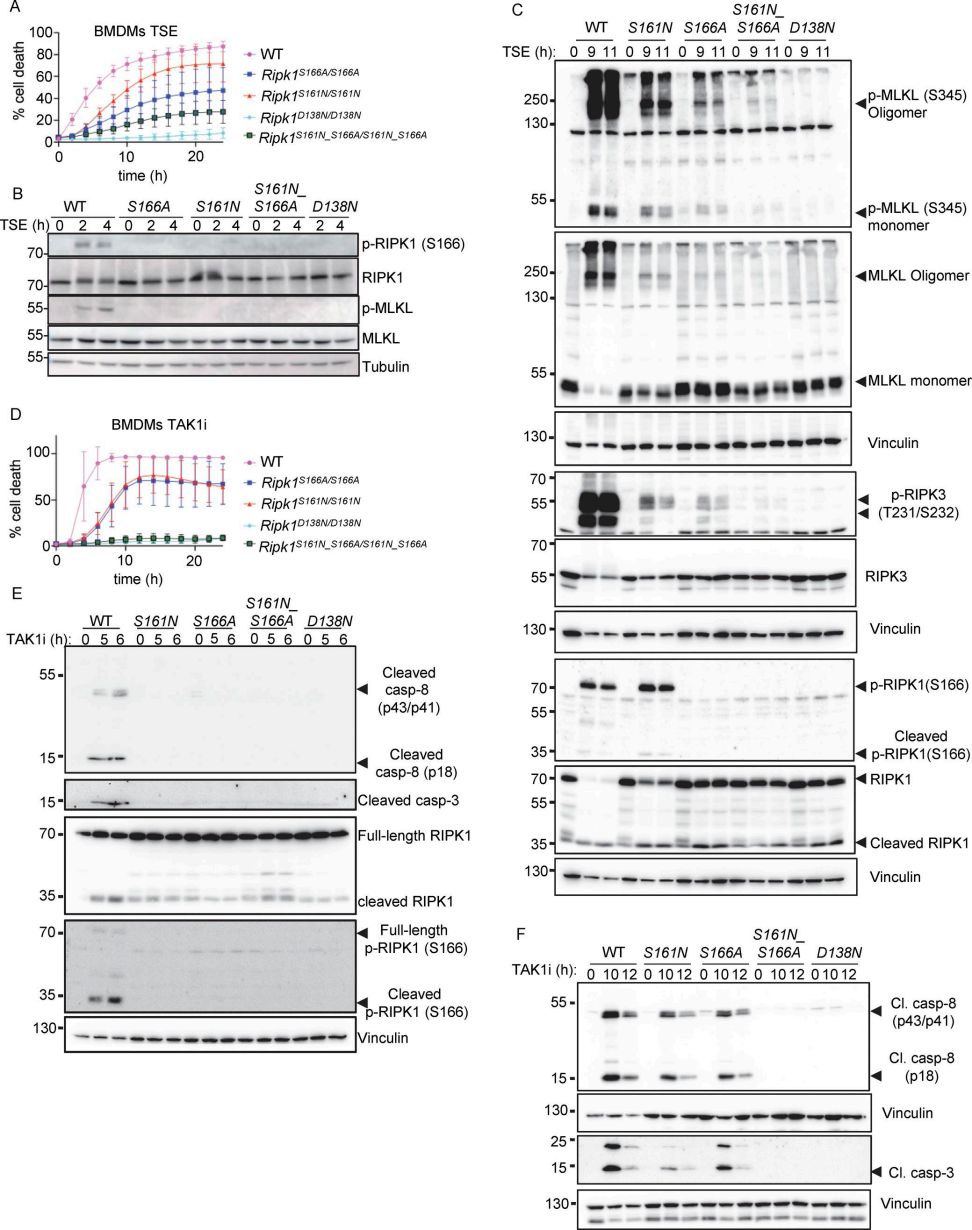
**Combined S161N_S166A mutation reveals partially redundant functions of S161 and S166 phosphorylation in RIPK1 kinase activity–dependent cell death. (A)** Graph depicting quantification of cell death in BMDMs from mice of the indicated genotypes treated with Emricasan (E, 5 µM), the SMAC mimetic compound birinapant (S, 1 µM), and TNF (T, 20 ng/ml). Graph shows the mean ± SEM of four independent experiments. **(B and C)** Immunoblots of BMDMs from mice of the indicated genotypes stimulated with TSE for 0, 2, and 4 h or for 0, 9, and 11 h. Representative of three independent experiments. **(D)** Graph depicting quantification of cell death in BMDMs from mice of the indicated genotypes treated with TAK1 inhibitor (TAK1i, 0.25 µM). Graph shows mean ± SEM of four independent experiments. **(E and F)** Immunoblots of BMDMs from mice of the indicated genotypes stimulated with TAK1i for 0, 5, and 6 h or for 0, 10, and 12 h. Representative of two independent experiments. Source data are available for this figure: SourceData F3.

Our studies in primary BMDMs showed that S161N mutation suppressed, but could not completely prevent RIPK1 kinase activity–dependent necroptosis and apoptosis, similarly to our previous findings in cells expressing RIPK1 with S166A mutation (Laurien et al., 2020). Since neither mutation alone could completely prevent RIPK1 kinase–dependent cell death in vitro, we reasoned that phosphorylation events on S161 and S166 may exhibit functional redundancy. To assess this hypothesis, we generated knock-in mice expressing RIPK1 with combined S161N and S166A mutations (*Ripk1*^S161N_S166A/S161N_S166A^) (Fig. S1, A and D). Indeed, side-by-side comparison revealed that *Ripk1*^S161N_S166A/S161N_S166A^ BMDMs were more strongly protected from TSE-induced necroptosis compared to *Ripk1*^S161N/S161N^ and *Ripk1*^S166A/S166A^ BMDMs (Fig. 3 A). Consistently, *Ripk1*^S161N_S166A/S161N_S166A^ BMDMs showed only very faint RIPK3 and MLKL phosphorylation as well as MLKL oligomerization at 9 or 11 h after TSE stimulation, which was weaker compared to *Ripk1*^S161N/S161N^ and *Ripk1*^S166A/S166A^ BMDMs (Fig. 3, B and C). Moreover, S166 phosphorylation was detected in *Ripk1*^S161N/S161N^ BMDMs 9 and 11 h after TSE stimulation (Fig. 3 C). Therefore, combined loss of S161 and S166 phosphorylation had an additive effect compared to the single mutations, resulting in strong suppression of TSE-induced necroptosis. Nevertheless, *Ripk1*^S161N_S166A/S161N_S166A^ BMDMs did show very low levels of necroptosis that were not observed in *Ripk1*^D138N/D138N^ BMDMs (Fig. 3, A–C), revealing that even the combined loss of both phosphorylation sites could not completely prevent RIPK1 kinase–induced necroptosis.

We then assessed how combined loss of S161 and S166 phosphorylation affected RIPK1-induced apoptosis. *Ripk1*^S161N_S166A/S161N_S166A^ BMDMs did not undergo apoptosis and did not show caspase-8 and caspase-3 cleavage in response to TAK1i treatment, similarly to *Ripk1*^D138N/D138N^ BMDMs, in contrast to *Ripk1*^S161N/S161N^ and *Ripk1*^S166A/S166A^ BMDMs, which showed delayed apoptosis and caspase activation under the same treatment (Fig. 3, D–F). Together, these results showed that autophosphorylation at S161 and S166 both contribute to RIPK1-induced cell death. While phosphorylation of both sites is required for full activation of RIPK1-mediated cell death, each site can partly compensate for the loss of the other to induce necroptosis and apoptosis. Interestingly, combined abrogation of autophosphorylation on S161 and S166 completely prevented TAK1i-induced apoptosis but could not completely suppress TSE-induced necroptosis, suggesting that additional phosphorylation sites on RIPK1 could compensate for the loss of S161 and S166 in inducing necroptosis.

**Figure 4.**
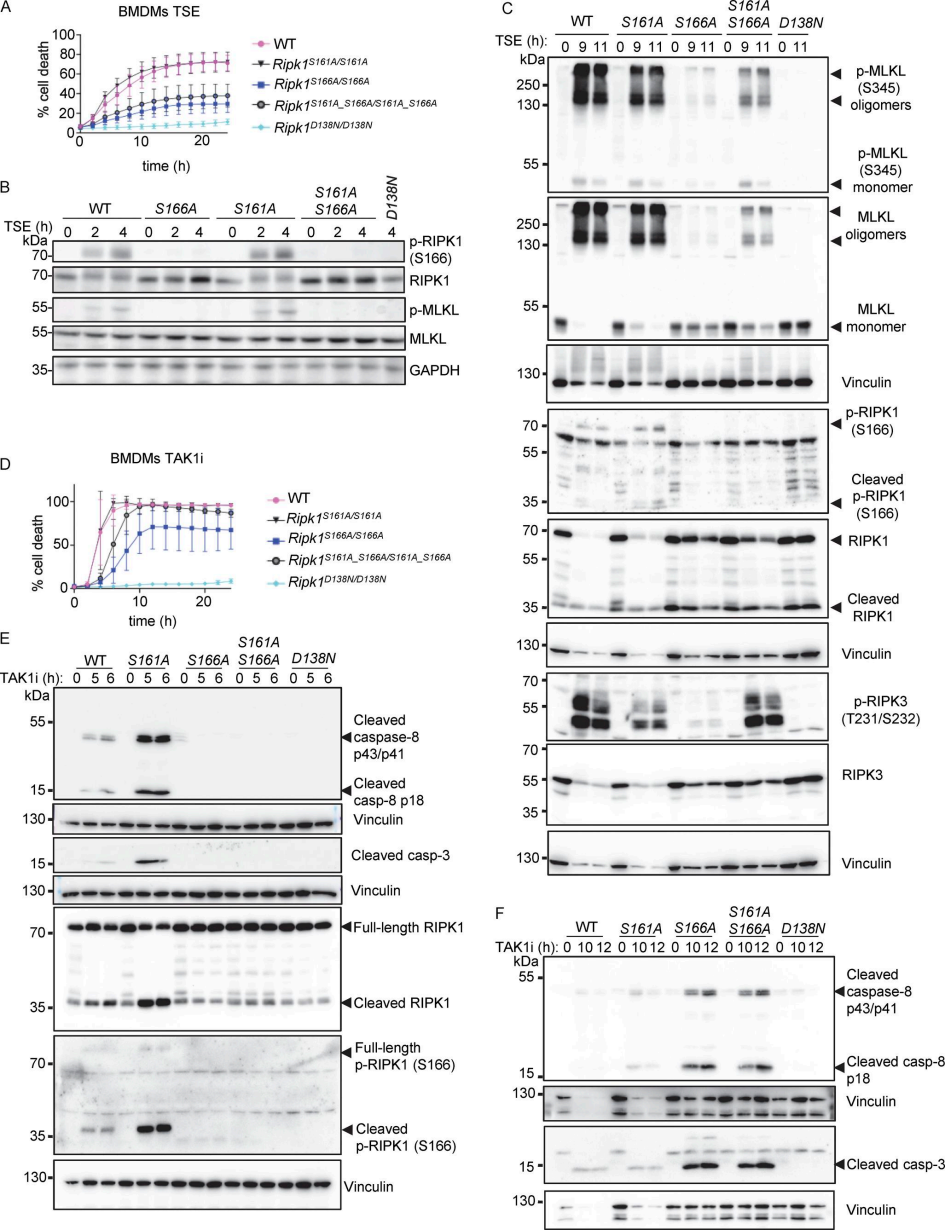
**S161A mutation partially counteracted the inhibitory effect of S166A mutation on RIPK1 kinase activity–dependent cell death. (A)** Graph depicting quantification of cell death in BMDMs from mice of the indicated genotypes treated with Emricasan (E, 5 µM), the SMAC mimetic compound birinapant (S, 1 µM), and TNF (T, 20 ng/ml). Graph shows the mean ± SEM of four independent experiments. **(B)** Immunoblots of BMDMs from mice of the indicated genotypes stimulated with TSE for 0, 2, and 4 h. Representative of three different experiments **(C)** Immunoblots of BMDMs from mice of the indicated genotypes stimulated with TSE for 0, 9, and 11 h. Representative of two independent experiments. **(D)** Graph depicting quantification of cell death in BMDMs from mice of the indicated genotypes treated with TAK1 inhibitor (TAK1i, 0.25 µM). Graph shows mean ± SEM of four independent experiments. **(E)** Immunoblots of BMDMs from mice of the indicated genotypes stimulated with TAK1i for 0, 5, and 6 h. Representative of two independent experiments. **(F)** Immunoblots of BMDMs from mice of the indicated genotypes stimulated with TAK1i for 0, 10, and 12 h. Representative of two independent experiments. Source data are available for this figure: SourceData F4.

Our previous results showing that S161A mutation did not inhibit RIPK1 kinase–dependent cell death are consistent with the notion that substitution of S161 with alanine might enforce an open conformation of RIPK1 due to loss of hydrogen bond formation with D156 (Zhang et al., 2017), thus making autophosphorylation on this site obsolete. We therefore asked whether substitution of S161 with alanine, by imposing an open conformation even in the absence of phosphorylation at this site, might be sufficient to overcome the inhibitory effect caused by loss of S166 phosphorylation. To assess this possibility, we generated knock-in mice expressing RIPK1 with combined S161A and S166A substitutions (*Ripk1*^S161A_S166A/S161A_S166A^) (Fig. S1 E). Mice harboring the S161A_S166A mutations were born at Mendelian ratio and did not show apparent abnormalities (data not shown). In response to TSE stimulation, BMDMs from *Ripk1*^S161A_S166A/S161A_S166A^ mice exhibited reduced cell death compared with *Ripk1*^S161A/S161A^ or WT cells but showed marginally increased cell death kinetics compared with *Ripk1*^S166A/S166A^ cells (Fig. 4 A). Consistently, TSE treatment induced RIPK3 and MLKL phosphorylation and MLKL oligomerization in *Ripk1*^S161A_S166A/S161A_S166A^ BMDMs, which was delayed and reduced compared with *Ripk1*^S161A/S161A^ cells but mildly enhanced compared with *Ripk1*^S166A/S166A^ cells after 9 and 11 h of stimulation (Fig. 4, B and C). In response to TAK1i treatment, *Ripk1*^S161A_S166A/S161A_S166A^ BMDMs showed accelerated cell death kinetics compared with *Ripk1*^S166A/S166A^ but delayed cell death compared with *Ripk1*^S161A/S161A^ BMDMs (Fig. 4 D). Consistently, *Ripk1*^S161A_S166A/S161A_S166A^ BMDMs showed delayed activation of caspase-8 and caspase-3 after TAK1i stimulation, similar to *Ripk1*^*S166A/S166A*^ BMDMs (Fig. 4, E and F). Therefore, *Ripk1*^S161A_S166A/S161A_S166A^ cells were more protected from necroptosis and apoptosis compared with *Ripk1*^S161A/S161A^ cells but showed slightly increased cell death compared with *Ripk1*^S166A/S166A^ cells. These results further support that S161A mutation imposes an open conformation on the RIPK1 activation loop that can partly overcome the inhibitory effect of S166A mutation. However, phosphorylation at S166 is required for full activation of RIPK1-mediated cell death also in the presence of the S161A mutation.

### Phosphomimetic S161E mutation bypasses the inhibitory effect of S166A in necroptosis induction

**Figure 5.**
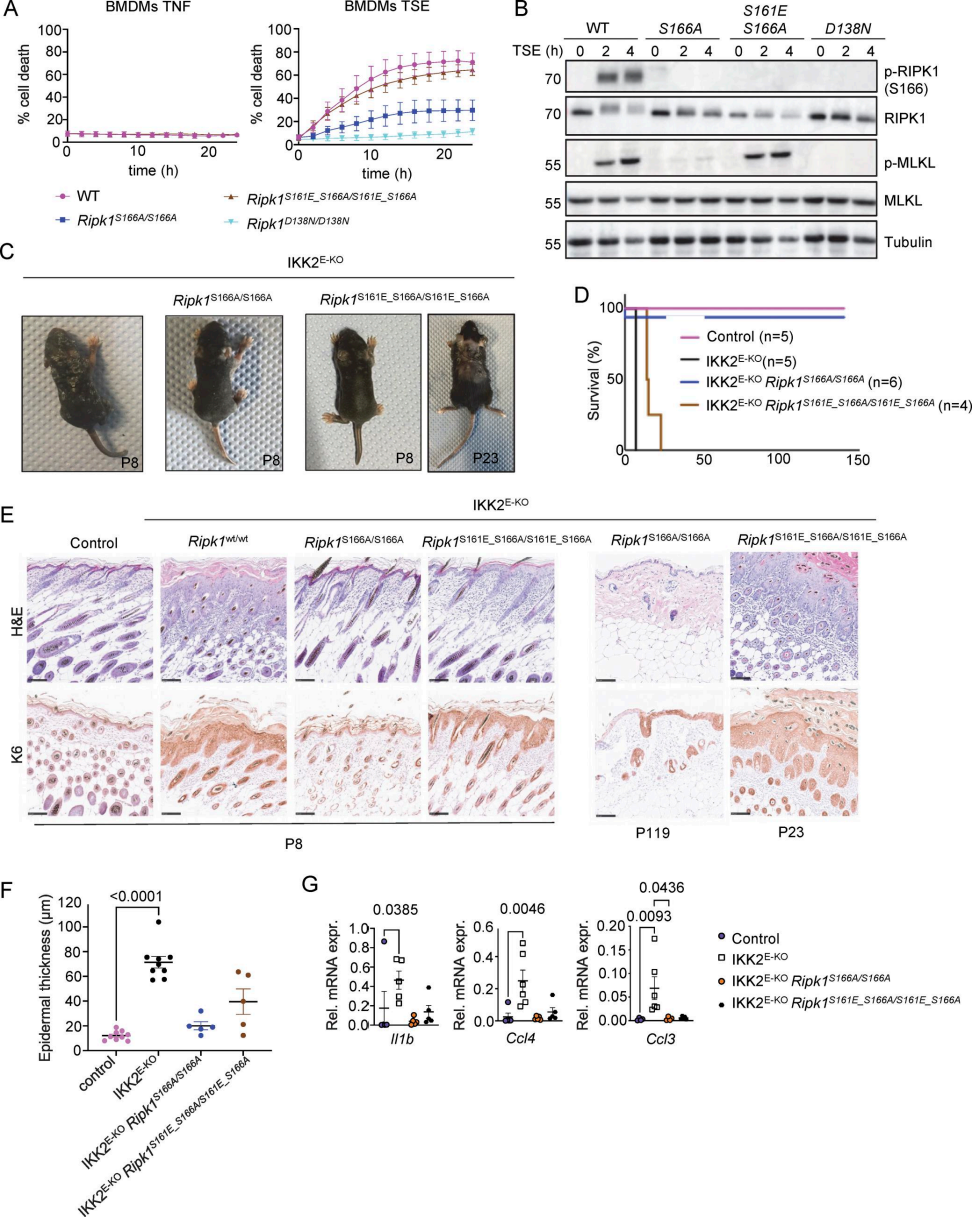
**Phosphomimetic S161E mutation is sufficient to confer necroptosis sensitivity in the absence of S166 phosphorylation. (A)** Graphs depicting quantification of cell death in BMDMs from mice of the indicated genotypes treated with TNF or with combinations of TNF (T, 20 ng/ml), Emricasan (E, 5 µM), and the SMAC mimetic compound birinapant (S, 1 µM). Graphs show mean ± SEM of at least three independent experiments. **(B)** Immunoblots of BMDMs from mice of the indicated genotypes stimulated with TSE for 0, 2, and 4 h. Representative of three independent experiments. **(C)** Representative photographs of IKK2^E-KO^ (*n* = 7), IKK2^E-KO^*Ripk1*^*S166A/S166A*^ (*n* = 6), and IKK2^E-KO^*Ripk1*^*S161E_S166A/S161E_S166A*^ (*n* = 9) mice. **(D)** Kaplan–Meier survival curve of mice with the indicated genotypes. **(E)** Representative images of sections from back skin of mice with the indicated genotypes stained with H&E or immunostained for K6 (scale bars = 100 µm; control *n* = 7, IKK2^E-KO^*n* = 7, IKK2^E-KO^*Ripk1*^*S166A/S166A*^*n* = 4, IKK2^E-KO^*Ripk1*^*S161E_S166A/S161E_S166A*^*n* = 4) at P8, P23, and P119. **(F)** Graph depicting epidermal thickness of mice with the indicated genotypes. Each dot represents one mouse. Epidermal thickness was measured at P8. Mean ± SEM are shown. Statistical significance was determined using Kruskal–Wallis test. **(G)** Graphs depicting relative mRNA expression of the indicated cytokines in RNA from whole-skin tissue of mice of the indicated genotypes measured by qRT-PCR. Each dot represents one mouse. Mean ± SEM are shown. Statistical significance was determined using Kruskal–Wallis test. Control mice include *Ikk2*^FL/FL^*K14Cre*^WT/WT^ or *Ikk2*^FL/WT^*K14Cre*^Tg/WT^ littermates with WT or mutant *Ripk1* alleles. Source data are available for this figure: SourceData F5.

Our results revealed that RIPK1 autophosphorylation at both S161 and S166 contribute to RIPK1 kinase activation and cell death induction in a partially redundant fashion. Previous studies using in vitro reconstitution and overexpression approaches suggested that autophosphorylation at S161 is sufficient for cell death induction (Zhang et al., 2017). We therefore wondered whether S161 phosphorylation could bypass the lack of S166 phosphorylation to induce RIPK1-dependent cell death. To this end, we generated mice expressing RIPK1 with a phosphomimetic serine to glutamic acid mutation at position 161 (S161E) in combination with the S166A mutation (*Ripk1*^S161E_166A/S161E_S166A^). *Ripk1*^S161E_166A/S161E_S166A^ mice were born at the expected Mendelian ratio and reached adulthood without showing apparent abnormalities, showing that the phosphomimetic mutation at S161 did not induce spontaneous pathology in vivo. BMDMs from *Ripk1*^S161E_166A/S161E_S166A^ mice that were left unstimulated or were treated with TNF alone did not undergo cell death (Fig. 5 A). In response to TSE stimulation, BMDMs from *Ripk1*^S161E_166A/S161E_S166A^ mice showed increased cell death compared with *Ripk1*^S166A/S166A^ cells, with cell death kinetics largely similar to those observed in WT BMDMs (Fig. 5 A). Consistent with the cell death assay results, we detected strong TSE-induced phosphorylation of MLKL in *Ripk1*^S161E_166A/S161E_S166A^ BMDMs compared with very weak MLKL phosphorylation in *Ripk1*^S166A/S166A^ cells (Fig. 5 B). Together, these results showed that phosphomimetic mutation on S161 could bypass the inhibitory effect of the absence of S166 phosphorylation in inducing necroptosis.

To study whether mimicking phosphorylation at S161 could promote necroptosis in the absence of S166 phosphorylation also in vivo, we assessed skin lesion development in IKK2^E-KO^*Ripk1*^S166A/S166A^ and IKK2^E-KO^*Ripk1*^S161E_S166A/S161E_S166A^ mice. In contrast to IKK2^E-KO^ mice that developed severe skin inflammation, necessitating their humane sacrifice at P8, IKK2^E-KO^*Ripk1*^S166A/S166A^ mice reached adulthood without showing skin lesions, with only two out of six mice showing mild focal upregulation of K6 in the epidermis at the age of 4–6 mo (Fig. 5, C–G). Thus, S166A mutation strongly protected IKK2^E-KO^ mice from necroptosis-induced skin inflammation similarly to the kinase-inactive D138N mutation (Kumari et al., 2021). IKK2^E-KO^*Ripk1*^S161E_S166A/S161E_S166A^ mice showed only minor signs of skin lesions at P8; however, these lesions progressed rapidly reaching the severity endpoint between 2 and 3 wk of age (Fig. 5, C–E). Therefore, phosphomimetic S161E mutation could bypass the inhibitory effect of S166A to induce keratinocyte necroptosis and skin inflammation in IKK2^E-KO^ mice, although with slower kinetics compared with WT RIPK1. Taken together, these results showed that S161 phosphorylation promotes RIPK1-mediated necroptosis in vitro and in vivo, also in the absence of S166 phosphorylation.

### Impaired apoptosis induction in cells expressing RIPK1 with S161E_S166A mutation

**Figure 6.**
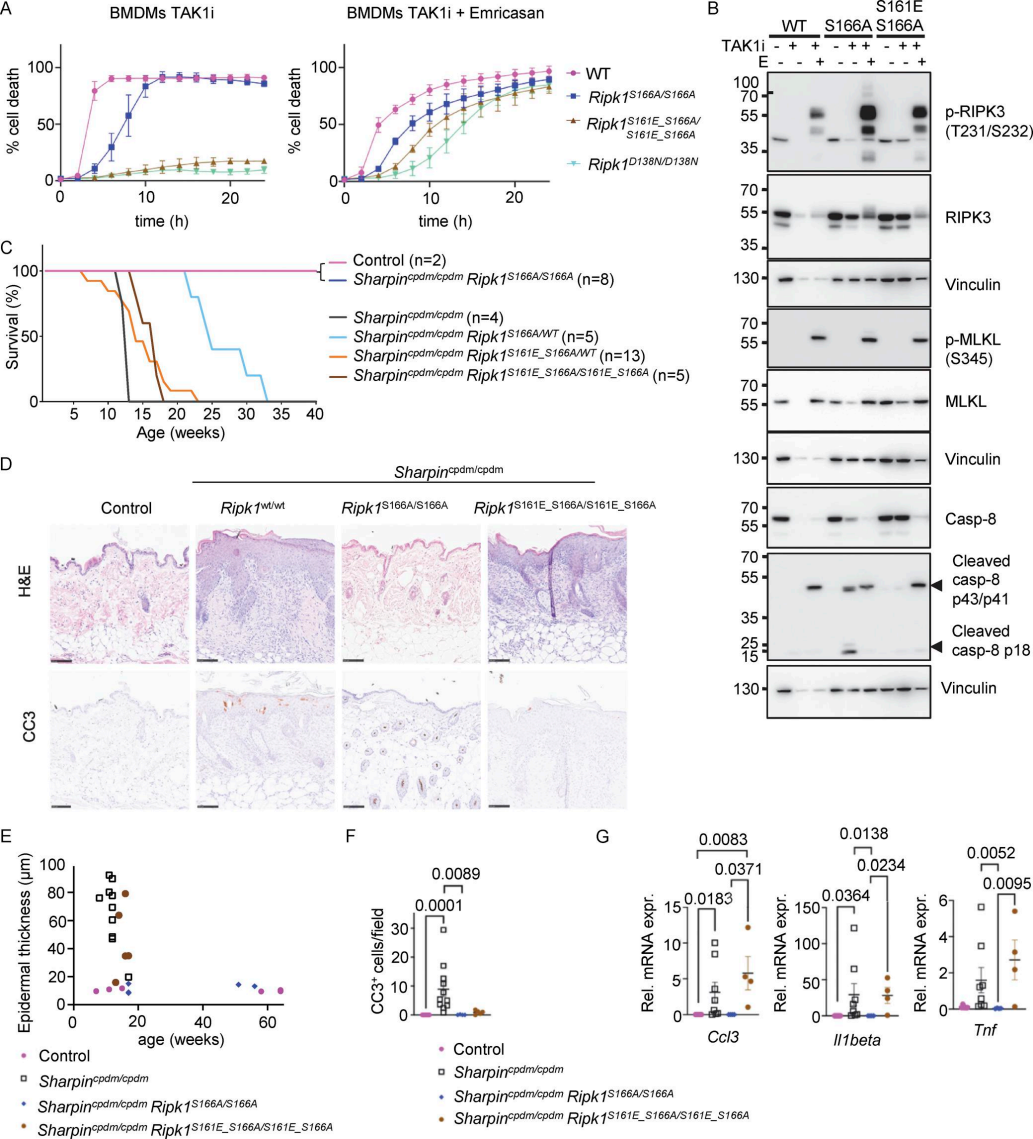
**Phosphomimetic S161E mutation does not sensitize cells to RIPK1 kinase activity–dependent apoptosis in the absence of S166 phosphorylation. (A)** Graphs depicting quantification of cell death in BMDMs from mice of the indicated genotypes treated with TAK1 inhibitor (TAK1i, 0.25 µM) alone and with a combination of TAK1i and Emricasan (5 µM). Graphs show mean ± SEM of three independent experiments. **(B)** Immunoblots of BMDMs from mice of the indicated genotypes stimulated with TAK1i with or without Emricasan (E, 5 µM). Representatives of two independent experiments. **(C)** Kaplan–Meier survival curve of mice with the indicated genotypes. **(D)** Representative images of sections from back skin of mice with the indicated genotypes stained with H&E or immunostained for CC3 (scale bars = 100 µm; control *n* = 5, *Sharpin*^*cpdm/cpdm*^*n* = 10, *Sharpin*^*cpdm/cpdm*^*Ripk1*^*S166A/S166A*^*n* = 4, *Sharpin*^*cpdm/cpdm*^*Ripk1*^*S161E_S166A/S161E_S166A*^*n* = 4). **(E)** Graph depicting measurement of epidermal thickness of mice with the indicated genotypes. Each dot represents one mouse. **(F)** Graph showing the average amount of CC3^+^ cells per optical field in mice with the indicated genotypes. Mean ± SEM are shown. Each dot represents one mouse. Statistical significance was determined using Kruskal–Wallis test. **(G)** Graphs depicting relative mRNA expression of the indicated cytokines in RNA from whole-skin tissue of mice of the indicated genotypes measured by qRT-PCR. Each dot represents one mouse. Means ± SEM are shown. Statistical significance was determined using Kruskal–Wallis test. Control mice include *Sharpin*^cpdm/WT^ or *Sharpin*^WT/WT^ littermates with WT or mutant *Ripk1* alleles. Source data are available for this figure: SourceData F6.

To test whether the phosphomimetic mutation on S161 could compensate for the inhibitory effect of S166A mutation in apoptosis induction, we measured TAK1i-induced cell death in BMDMs from WT, *Ripk1*^S166A/S166A^, and *Ripk1*^S161E_S166A/S161E_S166A^ mice. Surprisingly, *Ripk1*^S161E_S166A/S161E_S166A^ cells were as resistant as *Ripk1*^D138N/D138N^ BMDMs to TAK1 inhibitor treatment, whereas *Ripk1*^S166A/S166A^ BMDMs showed delayed death compared with WT cells under the same conditions (Fig. 6 A). Consistent with the cell death assay results, *Ripk1*^S161E_S166A/S161E_S166A^ BMDMs showed impaired caspase-8 processing in response to treatment with TAK1i alone compared with *Ripk1*^S166A/S166A^ cells (Fig. 6 B). However, *Ripk1*^S161E_S166A/S161E_S166A^ BMDMs underwent necroptosis in response to stimulation with TAK1i together with Emricasan, albeit with slightly reduced cell death kinetics compared with *Ripk1*^S166A/S166A^ BMDMs (Fig. 6, A and B). Notably, under these conditions also *Ripk1*^D138N/D138N^ cells showed delayed death, suggesting that TAK1i + Emricasan induces necroptosis by RIPK1 kinase–independent mechanisms at later time points (Fig. 6 A). To study whether the phosphomimetic mutation on S161 could compensate for the loss of S166 phosphorylation in driving caspase-8–dependent skin inflammation in vivo, we crossed the *Ripk1*^S161E_S166A/S161E_S166A^ mice into the *Sharpin*^cpdm/cpdm^ background. As we reported previously (Laurien et al., 2020), *Sharpin*^cpdm/cpdm^*Ripk1*^S166A/S166A^ mice did not develop skin lesions at least until the age of 40 wk (Fig. 6 C), showing that ablation of S166 phosphorylation could prevent the RIPK1 kinase activity–dependent keratinocyte death and skin pathology of *Sharpin*^cpdm/cpdm^ mice. In contrast, *Sharpin*^cpdm/cpdm^*Ripk1*^S161E_S166A/S161E_S166A^ mice developed skin lesions with kinetics largely similar to *Sharpin*^cpdm/cpdm^ mice (Fig. 6, C–G). Therefore, the phosphomimetic S161E mutation could bypass the requirement for S166 phosphorylation for the induction of RIPK1 kinase–dependent keratinocyte death, causing skin inflammation in vivo in the *Sharpin*^cpdm/cpdm^ mouse model. Interestingly, the skin lesions in *Sharpin*^cpdm/cpdm^*Ripk1*^S161E_S166A/S161E_S166A^ mice showed a strong reduction in the number of CC3^+^ cells in the epidermis compared with *Sharpin*^cpdm/cpdm^ (Fig. 6 F), suggesting that keratinocytes in these animals do not undergo apoptosis but may switch to necroptosis.

### Autophosphorylation at S161 and S166 are required for RIPK1 kinase activation

**Figure 7.**
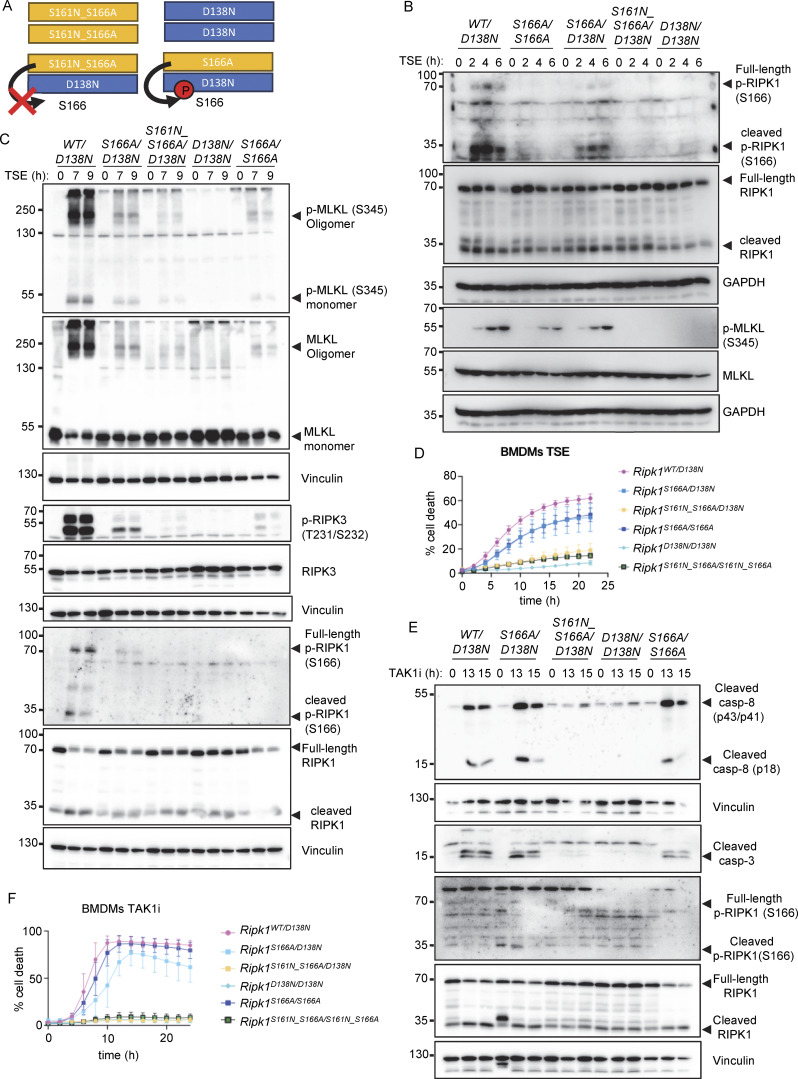
**S161 and S166 cooperate to regulate RIPK1 kinase activation. (A)** Schematic depicting the possible combinations of RIPK1 dimers in *Ripk1*^*S161N_S166A/D138N*^ and in *Ripk1*^*S166A/D138N*^ mice. Residue 166 on RIPK1 is labeled. **(B)** Immunoblots of BMDMs from mice of the indicated genotypes stimulated with TNF (T, 20 ng/ml), the SMAC mimetic compound birinapant (S, 1 µM), and Emricasan (E, 5 µM) (TSE) for 0, 2, 4, and 6 h. Representative of three independent experiments. **(C)** Immunoblots of BMDMs from mice of the indicated genotypes stimulated with TSE for 0, 7, or 9 h. Representative of two independent experiments. **(D)** Graph depicting quantification of cell death in BMDMs from mice of the indicated genotypes treated with TSE. Graph shows the mean ± SEM of seven independent experiments. **(E)** Immunoblots of BMDMs from mice of the indicated genotypes treated with TAK1 inhibitor (TAK1i, 0.25 µM) for 0, 13, and 15 h. Representative of two independent experiments. **(F)** Graph depicting quantification of cell death in BMDMs from mice of the indicated genotypes treated with TAK1i (0.25 µM). Graph shows the mean ± SEM of four independent experiments. Source data are available for this figure: SourceData F7.

Our results showed that autophosphorylation events on S161 and S166 are critical for the activation of RIPK1 kinase–dependent necroptosis and apoptosis. These autophosphorylation sites reside in the activation loop of RIPK1, and therefore mutations on these sites may inhibit its catalytic activity. Indeed, our previous studies in BMDMs from heterozygous *Ripk1*^D138N/S166A^ mice, in which the capacity of RIPK1-S166A to trans-autophosphorylate the kinase-inactive RIPK1-D138N can be measured by immunoblotting with antibodies against phosphorylated S166, showed that RIPK1-S166A exhibits reduced kinase activity (Laurien et al., 2020). Based on our findings that combined S161N_S166A mutation suppressed RIPK1 kinase–dependent necroptosis and apoptosis more strongly compared with the single mutations, we hypothesized that autophosphorylation at S161 may partially compensate for loss of S166 phosphorylation in promoting RIPK1 kinase activity (Fig. 7 A). To address this hypothesis, we generated *Ripk1*^S161N_S166A/D138N^ mice that express RIPK1-S161N_S166A from one allele and RIPK1-D138N from the other allele. In these cells, S166 phosphorylation can only occur if RIPK1-S161N_S166A phosphorylates RIPK1-D138N in trans (Fig. 7 A). Therefore, immunoblotting for phosphorylated S166 provides a readout of the kinase activity of RIPK1-S161N_S166A (Fig. 7 A). Since this approach ensures physiological endogenous expression levels, it provides an ideal experimental system to measure RIPK1 kinase activity in vivo that is superior to in vitro kinase assays or cellular systems based on overexpression of the mutated proteins. We therefore assessed cell death and S166 phosphorylation in these cells in response to TSE stimulation. *Ripk1*^WT/D138N^ BMDMs, which we used as positive control, showed S166 phosphorylation after TSE treatment (Fig. 7, B and C). Consistent with our previous studies, TSE-induced S166 phosphorylation was reduced but clearly detectable in *Ripk1*^S166A/D138N^ cells (Fig. 7, B and C). While we could not detect S166 phosphorylation in *Ripk1*^S161N_S166A/D138N^ BMDMs stimulated with TSE under the same conditions (Fig. 7, B and C), these cells showed weak phosphorylation and oligomerization of MLKL and cell death induction (Fig. 7, C and D). Therefore, abrogation of autophosphorylation on both S161 and S166 strongly suppressed but could not fully prevent RIPK1 kinase activation and necroptosis induction after TSE treatment. However, *Ripk1*^S161N_S166A/D138N^ BMDMs were fully protected from caspase-8 and caspase-3 cleavage and cell death after stimulation with TAK1i, in contrast to *Ripk1*^S166A/D138N^ cells, which showed weak activation of caspases and apoptosis (Fig. 7, E and F). Taken together, these results showed that autophosphorylation on both S161 and S166 contributes to RIPK1 kinase activation, with each site capable of partially compensating for the loss of the other.

## Discussion

RIPK1 is widely recognized as a kinase with an important role in cell death and inflammation and has been implicated in disease pathogenesis. While RIPK1 was initially reported to phosphorylate other substrates such as DRP1 (Wang et al., 2014), autophosphorylation is currently considered the main and critical function of RIPK1 kinase activity. Autophosphorylation is thought to promote conformational changes facilitating the interaction of RIPK1 with FADD and RIPK3 and the induction of caspase-8– and MLKL-mediated cell death. However, the functional significance of RIPK1 autophosphorylation on distinct sites as well as the potential hierarchies and cross talk between these sites remain largely unknown. Our results using knock-in mice expressing RIPK1 with mutations in S161 and S166 alone or in combination revealed important functions of these autophosphorylation sites in RIPK1-mediated cell death and inflammation in vitro and in vivo.

Our findings in *Ripk1*^S161N/S161N^ mice and cells showed that S161 phosphorylation is important for RIPK1-dependent MLKL-mediated necroptosis as well as for caspase-8–mediated apoptosis in vitro and in vivo. S161N mutation could prevent TNFR1-mediated keratinocyte necroptosis and inflammation in IKK2^E-KO^ mice, as well as TNFR1-mediated keratinocyte apoptosis and inflammation in *Sharpin*^cpdm/cpdm^ mice, revealing a critical physiological role of S161 phosphorylation for RIPK1-mediated cell death in vivo. However, S161N mutation only partially suppressed TNFR1-mediated necroptosis and apoptosis in cultured BMDMs, as opposed to complete protection offered by the catalytically inactive D138N mutation. Overall, the effects of S161N mutation were very similar to the effects of S166A mutation, suggesting that these two sites might act together in a functionally redundant fashion to promote RIPK1-mediated cell death responses. To address potential functional redundancies, we generated and analyzed mice with combined S161N_S166A mutations on RIPK1. *Ripk1*^S161N_S166A/S161N_S166A^ BMDMs were more strongly protected from TSE-induced necroptosis compared with the single mutants but did show weak activation of MLKL and low levels of cell death in some experiments. In contrast, *Ripk1*^S161N_S166A/S161N_S166A^ BMDMs were completely protected from TAK1 inhibitor–mediated activation of caspase-8–dependent cell death, suggesting that autophosphorylation on these sites is obligatory for caspase-8 activation. Together, these results showed that both S161 and S166 are important for RIPK1-induced cell death, with phosphorylation of both sites required for full activation of RIPK1-mediated cell death. Interestingly, combined abrogation of autophosphorylation on S161 and S166 completely prevented TAK1i-induced apoptosis but could not completely suppress TSE-induced necroptosis, suggesting that additional phosphorylation sites on RIPK1, such as T169 (Jetton et al., 2024; Laurien et al., 2020), could compensate for the loss of S161 and S166 in TSE-induced necroptosis.

Autophosphorylation on S161 or S166 could trigger RIPK1-mediated cell death directly by imposing conformational changes facilitating the recruitment and activation of downstream effectors such as RIPK3 and caspase-8, or indirectly by regulating RIPK1 kinase activation. Our experiments assessing RIPK1 kinase activity by detecting S166 phosphorylation of kinase-inactive RIPK1-D138N in heterozygous cells revealed that the capacity of the individual mutations on S161 and S166 to suppress RIPK1-dependent cell death correlated with their effect on the kinase activity of RIPK1. Our data suggest an interplay of autophosphorylation events at S161 and S166 in the RIPK1 activation loop, which are critical for full activation of its catalytic activity. Kinase activation by autophosphorylation in the activation loop is a well-documented activation mechanism in eukaryotic protein kinases that requires catalysis of an autoactivating reaction by an enzymatically inactive protein. This step is often achieved by assembly of inactive kinases into dimers and alignment of certain motifs, which allows for “one-hit” activating autophosphorylation reactions (Beenstock et al., 2016). Individual mutation of S161 or S166 partially inhibited, while combined mutation of both sites nearly completely suppressed RIPK1 kinase activity and cell death induction, suggesting that autophosphorylation at these sites is critical for full activation of RIPK1 and induction of downstream cell death signaling. Autophosphorylation is thought to impose conformational changes on RIPK1 facilitating its interaction with downstream cell death effectors and the formation of cell death–inducing signaling complexes. Overall, the decision whether cell death is induced may depend on whether a certain threshold of kinase activity is reached to achieve autophosphorylation in a certain amount of RIPK1 molecules within a cell. Thus, lower levels of kinase activity in *Ripk1*^S166A/S166A^ and *Ripk1*^S161N/S161N^ would result in overall fewer cells reaching the threshold for cell death induction, which is sufficient to block RIPK1-mediated cell death and inflammation in vivo but can be bypassed by increasing the strength of the stimulus in cell culture experiments.

The finding that abrogating autophosphorylation on S161 or S166 strongly inhibited RIPK1 kinase activity makes it difficult to assess the role of specific autophosphorylation events in directly promoting RIPK1-mediated cell death induction by facilitating recruitment and activation of RIPK3 and/or caspase-8. To specifically assess whether S161 phosphorylation could be sufficient for RIPK1-mediated cell death, we employed *Ripk1*^S161E_S166A/S161E_S166A^ mice expressing RIPK1 with a phosphomimetic S161E mutation in the context of S166A mutation. Introducing a phosphomimetic S161E mutation was sufficient to overcome the inhibitory effect of the S166A mutation in the induction of TSE-induced necroptosis in vitro, as well as for keratinocyte necroptosis and skin inflammation in IKK2^E-KO^ mice in vivo. These experiments provided evidence that S161 phosphorylation could bypass the requirement for S166 phosphorylation for the induction of necroptosis. Interestingly, *Ripk1*^S161E_S166A/S161E_S166A^ BMDMs showed impaired caspase-8–mediated apoptosis in response to TAK1i treatment but readily succumbed to necroptosis induced by TAK1i + Emricasan. Moreover, *Sharpin*^cpdm/cpdm^*Ripk1*^S161E_S166A/S161E_S166A^ mice developed skin lesions with similar kinetics and severity as *Sharpin*^cpdm/cpdm^ expressing WT RIPK1, but had much less CC3^+^ cells in the epidermis compared with *Sharpin*^cpdm/cpdm^ animals, suggesting that keratinocytes in these mice do not undergo apoptosis but may switch to necroptosis. While the underlying molecular mechanism remains to be fully elucidated, these results indicated that S161E mutation specifically sensitizes cells to necroptosis.

Collectively, our results support a model in which an interplay of autophosphorylation events at S161 and S166 is required to promote kinase activation and the cell death–inducing function of RIPK1. While autophosphorylation in both S161 and S166 is required for full activation of RIPK1 and the induction of cell death, the two sites show a degree of functional redundancy, with mutation of either residue resulting in partial inhibition of RIPK1-mediated cell death in cell culture. However, under more physiological conditions in vivo, mutations abolishing autophosphorylation on either S161 or S166 were sufficient to prevent RIPK1-mediated cell death and inflammation. Our results are in agreement with an accompanying paper by Han et al. (2025), who report that RIPK1 autophosphorylation promotes TNF-induced necroptosis in mice. Therefore, S161 and S166 phosphorylation can serve as valuable biomarkers for the stratification of patients that might benefit most from treatment with RIPK1 kinase inhibitors that are currently under clinical development.

## Materials and methods

### Mice

*Ripk1* ^D138N/D138N^ (Polykratis et al., 2014), *Ripk1*^S166A/S166A^ (Laurien et al., 2020), *Sharpin*^cpdm/cpdm^ (Seymour et al., 2007), *Ikk2*^FL/FL^ (Pasparakis et al., 2002), *Caspase-1*^*−/−*^ (Van Gorp et al., 2016), and *K14Cre* (Hafner et al., 2004) mice were described previously. *Ripk1*^S161N/S161N^, *Ripk1*^S161A/S161A^, *Ripk1*^S161N_S166A/S161N_S166A^, *Ripk1*^S161A_S166A/S161A_S166A^, *Ripk1*^S161E_166A/S161E_S166A^, *Gsdmd*^*−/−*^, and *Gsdme*^*−/−*^ mice were generated in this study using CRISPR-Cas9–mediated gene targeting in zygotes from C57Bl/6N mice. Oocytes fertilized with sperm of *Ripk1*^*S166A/S166A*^ males were electroporated with the following knock-in oligos and Cas9 protein for the respective mouse lines (mutated codons are bolded):

5′-TAA​CTT​CCA​TTG​GCC​CCT​CTT​CTT​TTC​CAG​ATA​GCC​GAT​CTT​GGT​GTG​GCT**GCG**TTT​AAG​ACA​TGG**GCC**AAA​CTG​ACT​AAG​GAG​AAA​GAC​AAC​AAG​CAG​AAA​GAA​GTG​AGC​AGC​ACC​ACT-3′ (*Ripk1*^*S161A_S166A/S161A_S166A*^), 5′-CTT​AGT​GAT​AAC​TTC​CAT​TGG​CCC​CTC​TTC​TTT​TCC​AGA​TAG​CCG​ATC​TTG​GTG​TAG​CT**AAC**TTT​AAG​ACA​TGG**GCG**AAA​CTG​ACT​AAG​GAG​AAA​GAC​AAC​AAG​CAG​AAA​GAA​GTG​AGC​AGC​ACC​ACT​AAG​AAG​AAC-3′ (*Ripk1*^*S161N_S166A/S161N_S166A*^), and 5′-CTT​AGT​GAT​AAC​TTC​CAT​TGG​CCC​CTC​TTC​TTT​TCC​AGA​TAG​CCG​ATC​TTG​GTG​TGG​CT**GAG**TTT​AAG​ACA​TGG**GCG**AAA​CTG​ACT​AAG​GAG​AAA​GAC​AAC​AAG​CAG​AAA​GAA​GTG​AGC​AGC​ACC​ACT​AAG​AAG​AAC-3′ (*Ripk1*^*S161E_S166A/S161E_S166A*^).

Fertilized WT oocytes were electroporated with the following knock-in oligos and Cas9 protein for the respective mouse lines:

5′-TAA​CTT​CCA​TTG​GCC​CCT​CTT​CTT​TTC​CAG​ATA​GCC​GAT​CTT​GGT​GTG​GCT**GCG**TTT​AAG​ACA​TGG**AGC**AAA​CTG​ACT​AAG​GAG​AAA​GAC​AAC​AAG​CAG​AAA​GAA​GTG​AGC​AGC​ACC​ACT-3′ (*Ripk1*^*S161A/S161A*^) and 5′-CTT​AGT​GAT​AAC​TTC​CAT​TGG​CCC​CTC​TTC​TTT​TCC​AGA​TAG​CCG​ATC​TTG​GTG​TAG​CT**AAC**TTT​AAG​ACA​TGG​AGC​AAA​CTG​ACT​AAG​GAG​AAA​GAC​AAC​AAG​CAG​AAA​GAA​GTG​AGC​AGC​ACC​ACT​AAG​AAG​AAC-3′ (*Ripk1*^*S161N/S161N*^).

To generate *Gsdme*^*−/−*^ mice, one gRNA (Gsdme gRNA: 5′-TTT​CTG​GAC​ATG​CTG​GAT​GG-3′) targeting exon 7 was in vitro transcribed and coelectroporated into fertilized WT oocytes together with Cas9 protein. To generate *Gsdmd*^*−/−*^ mice, two gRNAs (Gsdmd gRNA1: 5′-GCA​GTA​TAC​ACA​CAT​TCA-3′ and Gsdmd gRNA2: 5′-GCG​TGT​GAC​TCA​GAA​GAC​CT-3′) targeting exon 3 were coelectroporated into fertilized WT oocytes together with Cas9 protein. After confirmation of the correct mutations by genomic DNA sequencing analysis, founder mice carrying the targeted mutations were backcrossed to C57BL/6N mice to establish independent mouse lines. Based on our previous experience in the analysis of the *Sharpin*^cpdm/cpdm^ and IKK2^E-KO^ mouse models of skin inflammation, we aimed to analyze at least five animals per group.

All mice were maintained at the specific pathogen–free animal facility of the Cologne Excellence Cluster for Aging and Aging-Associated Diseases (CECAD) Research Center of the University of Cologne at a temperature of 22 ± 2°C, 55 ± 5% relative humidity under a 12-h dark/light cycle, and given a regular chow diet (Harlan, diet number 2918) as well as water ad libitum. All animal procedures were conducted in accordance with European national and institutional guidelines, and all protocols were approved by the responsible local authorities (Landesamt für Natur, Umwelt und Verbraucherschutz Nordrhein-Westfalen, Germany). Animals requiring medical attention were provided with appropriate care. No other exclusion criteria existed. All mouse studies were performed in a blinded fashion, and animals were assigned randomly to groups.

### Primary cell isolation and culture

BMDMs were isolated from mice according to standard procedures. Bones were dissected and flushed with PBS; the collected bone marrow was spun down, plated, and maintained in Dulbecco’s modified eagle medium (Gibco) supplemented with penicillin-streptomycin, L-glutamine, sodium pyruvate, and HEPES buffer solution (1% each, all Gibco) and 10% fetal bovine serum superior (Biochrom) and 20 ng ml^−1^ M-CSF (RRID: 12343118; ImmunoTools). BMDMs were differentiated for 6 days before plating for experiments. BMDMs were kept at 37°C with 5% CO_2_.

### Histological analysis of tissue sections

Skin tissue samples were fixed overnight in 4% paraformaldehyde, embedded in paraffin, and cut in 3–5-µm sections. For histopathological analysis, paraffin sections were rehydrated using decreasing ethanol concentrations, and heat-induced antigen retrieval was performed. Sections were incubated with primary antibodies against cleaved caspase-3 (RRID: 9661; Cell Signaling; Lot: 47) and Keratin 6 (RRID: 905701; BioLegend, Lot: B379761). For visualization, the ABC Kit Vectastain Elite (Vector Laboratories) and DAB substrate (DAKO and Vector Laboratories) were used. Epidermal thickness was analyzed by measuring epidermal thickness in four optical fields per section with four measurements per field. For determination of CC3^+^ cell numbers, four optical fields per section at 10× magnification were counted, and the average was plotted. Quantification of CC3^+^ cells and epidermal thickness were performed in a blinded fashion. To compare the effects of the different RIPK1 mutations on the development of skin lesions, the same groups of *Sharpin*^*cpdm/cpdm*^ mice and respective littermate controls, as well as IKK2^E-KO^ mice and respective K14Cre-negative littermate controls, were used.

### Immunoblotting

BMDMs were seeded at 1,000,000 cells per well in a 6-well plate. After stimulation, cells were lysed in protein lysis buffer supplemented with protease and phosphatase inhibitors for 20 min on ice. Samples were heated at 95°C for 8 min. Protein lysates were separated using SDS gel electrophoresis and transferred to PVDF membranes (Cat# IPVH00010; Millipore). Membranes were blocked for 1 h in 5% milk/0.1% PBS-T and incubated overnight at 4°C with primary antibodies against RIPK1 (RRID: AB_397832, Cat# 610459; BD Biosciences, Lot: 9213795), RIPK1 (RRID: AB_2305314, Cat# 3493; Cell Signaling, Lot: 3), p-RIPK1(S166) (RRID: AB_2799000, Cat# 31122; Cell Signaling Technology, Lot: 6), p-RIPK3(T231/S232) (RRID: AB_2937060, Cat# 91702; Cell Signaling, Lot: 4), p-MLKL (S345) (RRID: AB_2799112, Cat# 37333; Cell Signaling, Lot: 4), MLKL (RRID: AB_2820284, Cat# MABC604; Millipore, Lot: 3887745), RIPK3 (RRID: AB_2722663, Cat# 15828; Cell Signaling, Lot: 1), Cleaved-Caspase-3 (RRID: AB_2341188, Cat# 9661; Cell Signaling, Lot: 47), Cleaved-Caspase-8 (RRID: AB_10891784, Cat# 8592; Cell Signaling, Lot: 6), Caspase-8 (RRID: AB_10545768, Cat# 4790; Cell Signaling, Lot: 4), GAPDH (RRID:AB_10077627, Cat# NB300-221; Novus Biologicals, Lot: 061424), Vinculin (RRID: AB_2728768, Cat# 13901; Cell Signaling, Lot: 9), Caspase-1 (RRID:AB_2490249, Cat# AG-20B-0042-C100; AdipoGen, Lot: A43682304), GSDMD (RRID: AB_2888940, Cat# ab219800; Abcam, Lot: GR3205112-17), GSDME (RRID: AB_2737000, Cat# ab215191; Abcam, Lot: GR3279425), and Tubulin (RRID: AB_477582, Cat# T6074; Sigma-Aldrich). Secondary antibodies used were anti-mouse IgG HRP-linked antibody (RRID: AB_772210, Cat# NA931; Cytiva, Lot: 18228250), anti-rat (RRID: AB_2338140, Cat# 112-035-175; Jackson ImmunoResearch Labs, Lot: 156796), and anti-rabbit IgG HRP-linked antibody (RRID: AB_772206, Cat# NA934V; Cytiva, Lot: 18258065). Signals were detected using SuperSignal West Pico Chemiluminescent substrate (Cat# 34080; Thermo Fisher Scientific), Amersham ECL Western Blotting Detection Reagent (GE Healthcare), or SuperSignal West Pico PLUS Chemiluminescent Substrate (Cat# 34580; Thermo Fisher Scientific) and SuperSignal West Femto (Cat# 34095; Thermo Fisher Scientific). The membranes were reprobed after incubation in Restore Western Blot stripping buffer (21059; Thermo Fisher Scientific) or after extensive washing for primary antibodies of different species.

### MLKL oligomerization detection

BMDMs were seeded at 1,000,000 cells per well in a 6-well plate. After stimulation, cells were lysed with nonreducing sample buffer (300 mM Tris-Cl [pH 6.8], 50% glycerol, 0.05% bromophenol blue, and 10% SDS) immediately. Total cell lysates were separated using SDS-PAGE, transferred to PVDF membrane (IPVH00010; Millipore), and detected with the indicated antibodies.

### Cell death assays

BMDMs were seeded at 30,000 cells per well in a 96-well plate. The following day, cells were pre-treated with combinations of Emricasan (S7775; Selleckchem) and Birinapant (2597; BioVision) and TAK1 inhibitor 5Z-7 Oxozeaenol (O9890; Sigma-Aldrich, 0.25 µM) in the presence of the dead cell stain DRAQ7 (7406; Cell Signaling, 0.15 µM) for 30 min before addition of recombinant mouse TNF (VIB Protein Service Facility, Ghent) or LPS (ALX-581-010-L002; Enzo, 100 ng*ml^−1^) or TAK1 inhibitor 5Z-7 Oxozeaenol (O9890; Sigma-Aldrich, 0.25 µM). Dead cells were imaged in real time for 24 h in intervals of 2 h via fluorescence signals using an IncuCyte S3 Live-Cell Analysis System (Essen Bioscience). Total cell numbers were calculated by treating three wells per genotype in each experiment with the cell-permeable fluorescent stain DRAQ5 (#4084L; Cell Signaling Technology, 500 nM) and imaging at a 2-h time point. Resulting images were analyzed using the software package of the IncuCyte, which allows quantification of the number of DRAQ7- or DRAQ5-positive cells. Dead cell counts acquired via DRAQ7 staining were divided by the total cell number of DRAQ5-positive cells to yield the percentage of cell death at each time point. Curves show the mean of at least three independent experiments.

### Quantitative RT-PCR (qRT-PCR)

Total RNA was extracted using a Direct-zol RNA isolation kit (Biozol). cDNA was prepared using the LUNA RT SuperMix kit (E3010L; NEB). All qRT-PCRs were performed with TaqMan probes (*Il1b* Mm00434228_m1, *Il6* Mm00446190_m1, *Tnf* Mm00443258_m1, *Ccl4* [Mm00443111_m1], and *Ccl3* [Mm00441258_m1], all Thermo Fisher Scientific). Relative expression of gene transcripts was analyzed using the 2^−ΛCt^ method, and *Tbp* (Mm00446973_m1) was used as a reference gene. The same *Sharpin*^*cpdm/cpdm*^ control mice and respective WT controls were used in all experiments for comparison. The same IKK2^E-KO^ control mice and respective WT controls were used in all experiments for comparison.

### Statistical analysis

Statistical analysis was performed with Prism 10 (GraphPad). All statistical details can be found in the figure legends or in the figures. Data shown in graphs display mean. No data were excluded.

### Online supplemental material

Fig S1 shows the targeting scheme as well as genomic DNA sequencing data for mice harboring RIPK1 mutations generated in this study. Fig S2 shows that the TAK1i-induced cell death in BMDMs requires autocrine TNF-TNFR1 signaling and depends on caspase-8 but occurs independently of caspase-1 and GSDMD and GSDME. Additionally, Fig S2 shows that the different RIPK1 mutations do not impact TNF + CHX-induced cell death in BMDMs. Fig S3 shows the targeting scheme and genomic DNA sequencing data for the generation of *Gsdme*^*−/−*^ and *Gsdmd*^*−/−*^ mice.

## Supplementary Material

SourceData F1 is the source file for Fig. 1.

SourceData F2 is the source file for Fig. 2.

SourceData F3 is the source file for Fig. 3.

SourceData F4 is the source file for Fig. 4.

SourceData F5 is the source file for Fig. 5.

SourceData F6 is the source file for Fig. 6.

SourceData F7 is the source file for Fig. 7.

SourceData FS2 is the source file for Fig. S2.

## Data Availability

The datasets generated during and/or analyzed during the current study are available from the corresponding author on reasonable request.
