# Supplementary figures and images for: RIPK1 autophosphorylation at S161 mediates cell death and inflammation
Source: J Exp Med. 2025 Sep 25;222(12):e20250279. doi: 10.1084/jem.20250279 (PMC12462663; doi:10.1084/jem.20250279)

Figure 1B

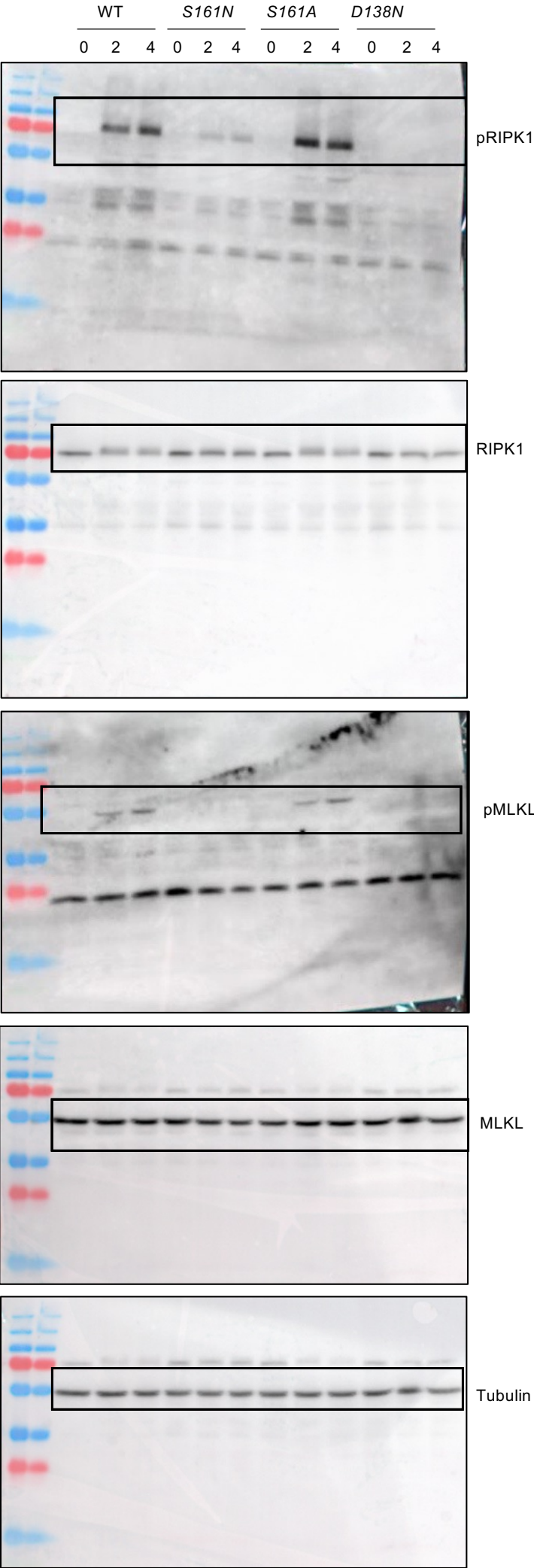

Supplement: SourceData F1 — is the source file for Fig. 1. [file jem_20250279_sourcedataf1.pdf]

Figure 2B

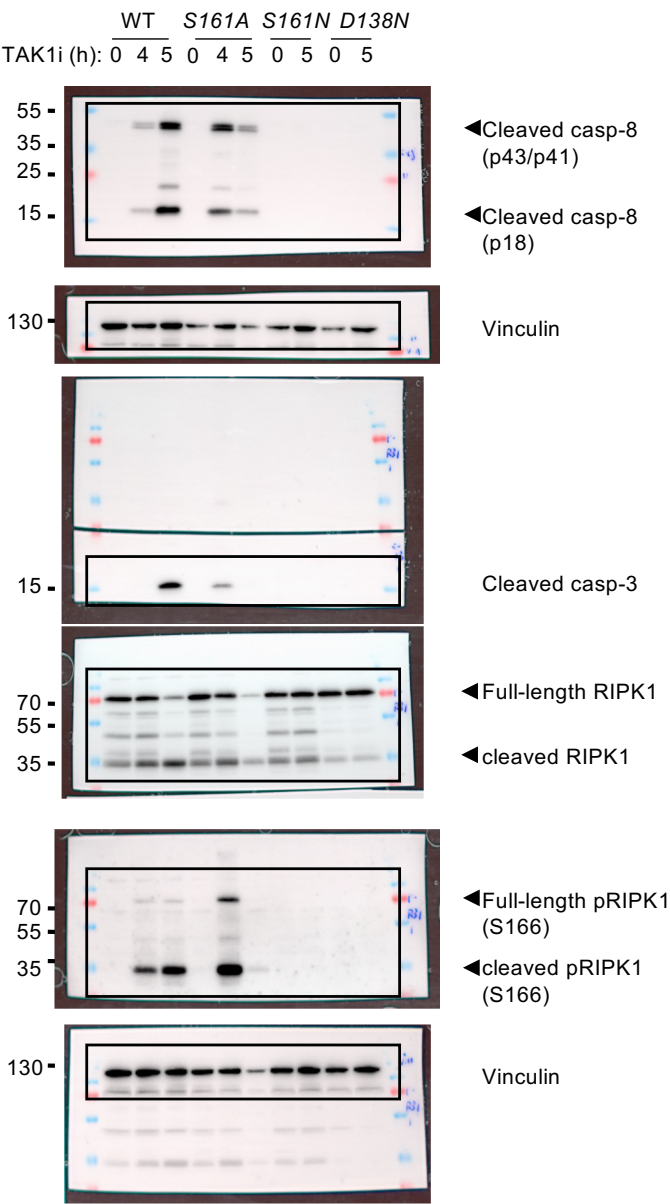

Supplement: SourceData F2 — is the source file for Fig. 2. [file jem_20250279_sourcedataf2.pdf]

Figure 3B

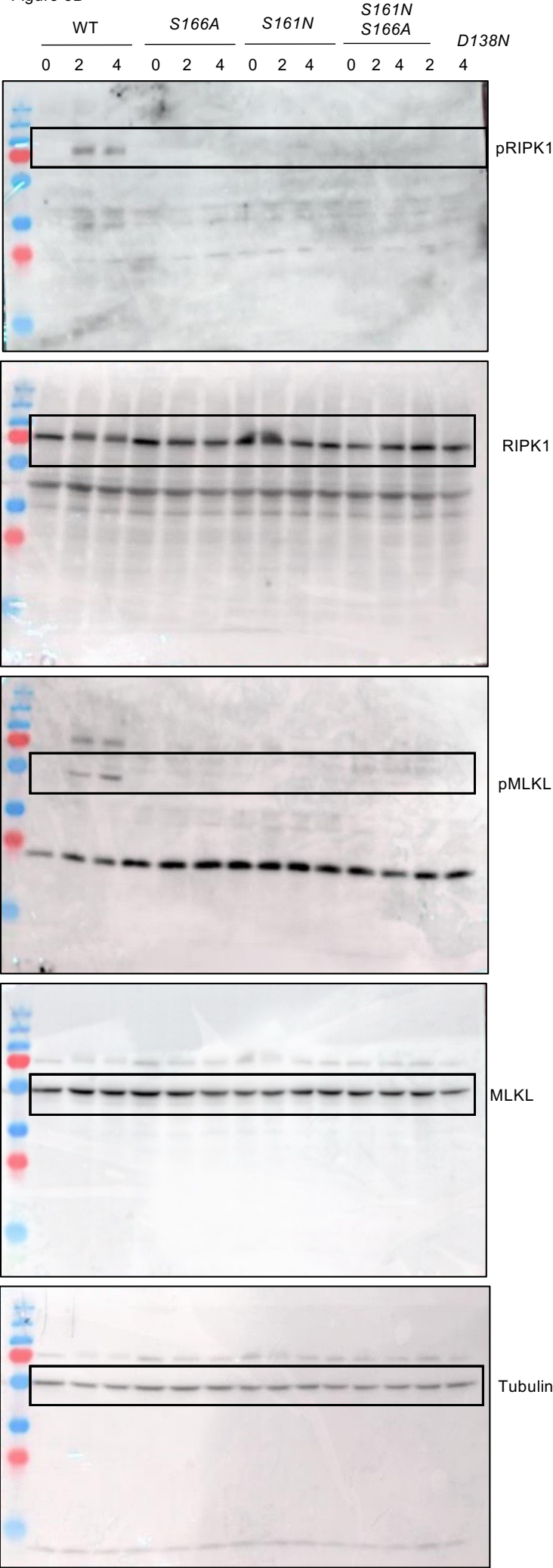

Figure 3C

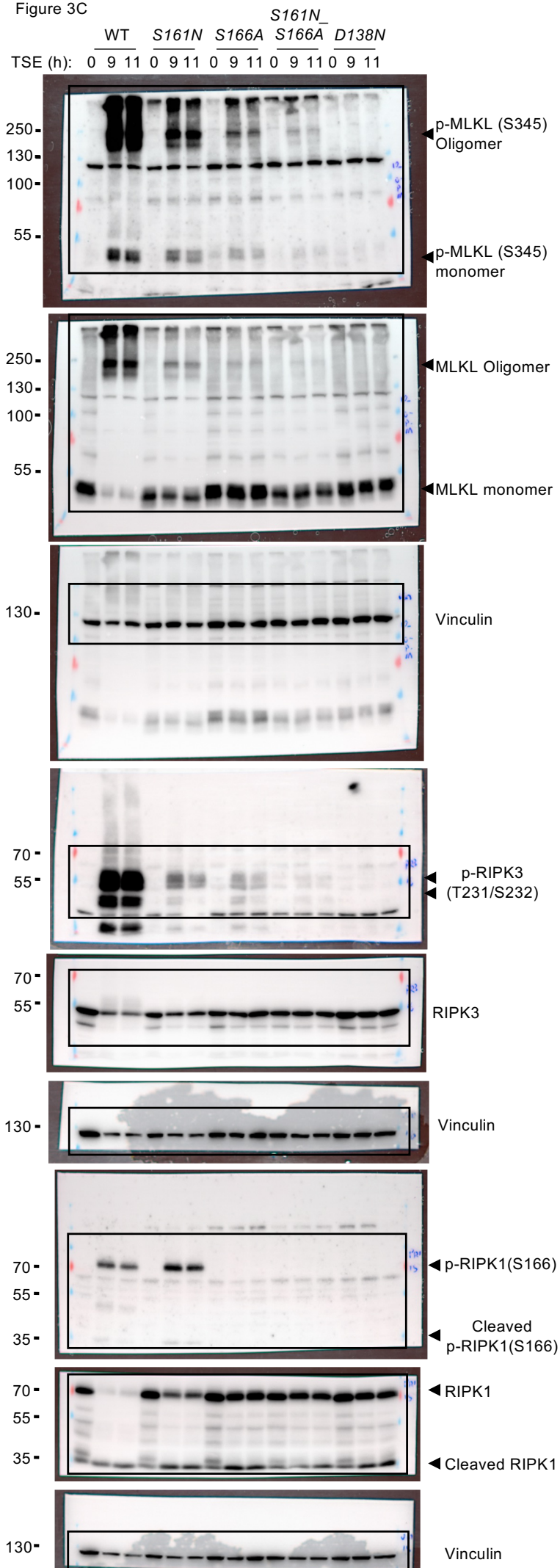

Figure 3E

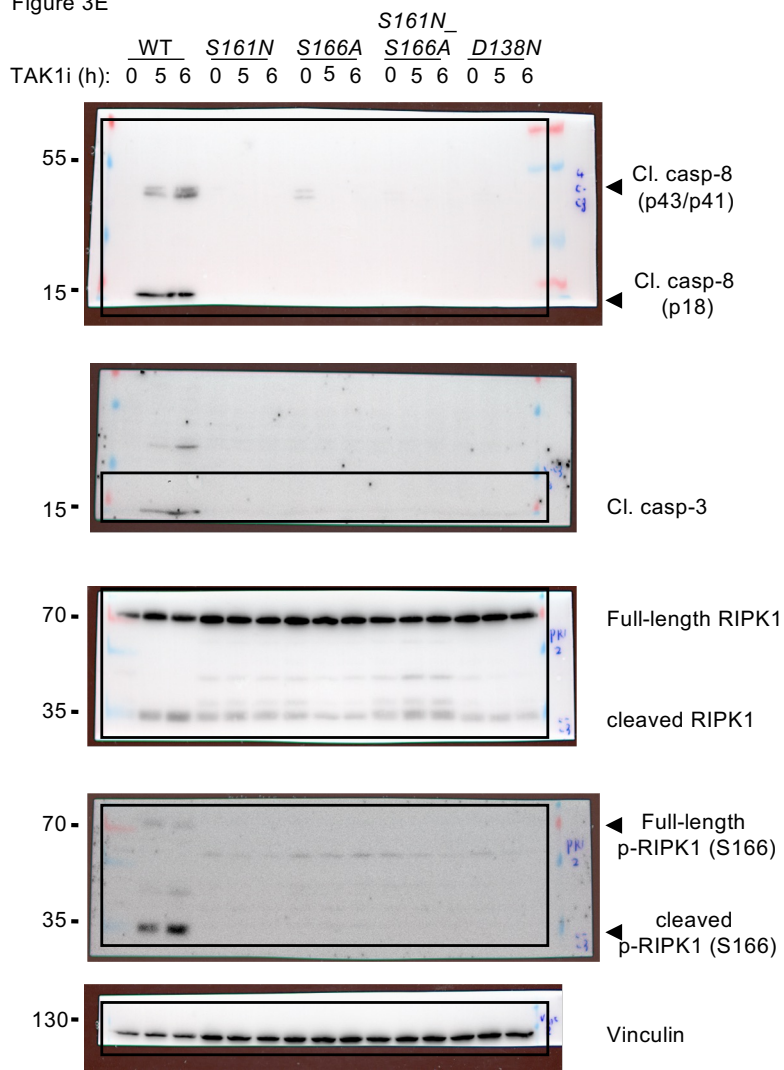

Figure 3F

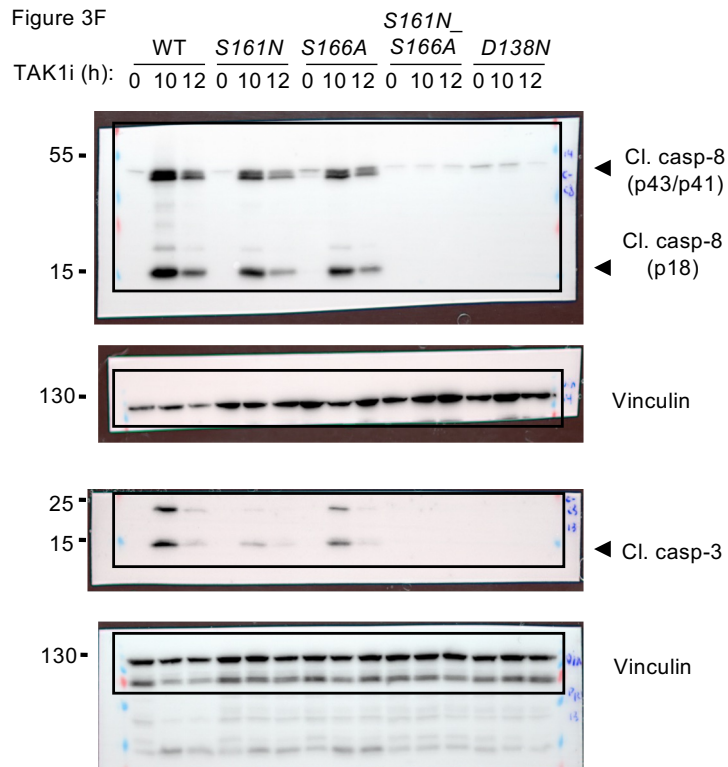

Supplement: SourceData F3 — is the source file for Fig. 3. [file jem_20250279_sourcedataf3.pdf]

Figure 4B

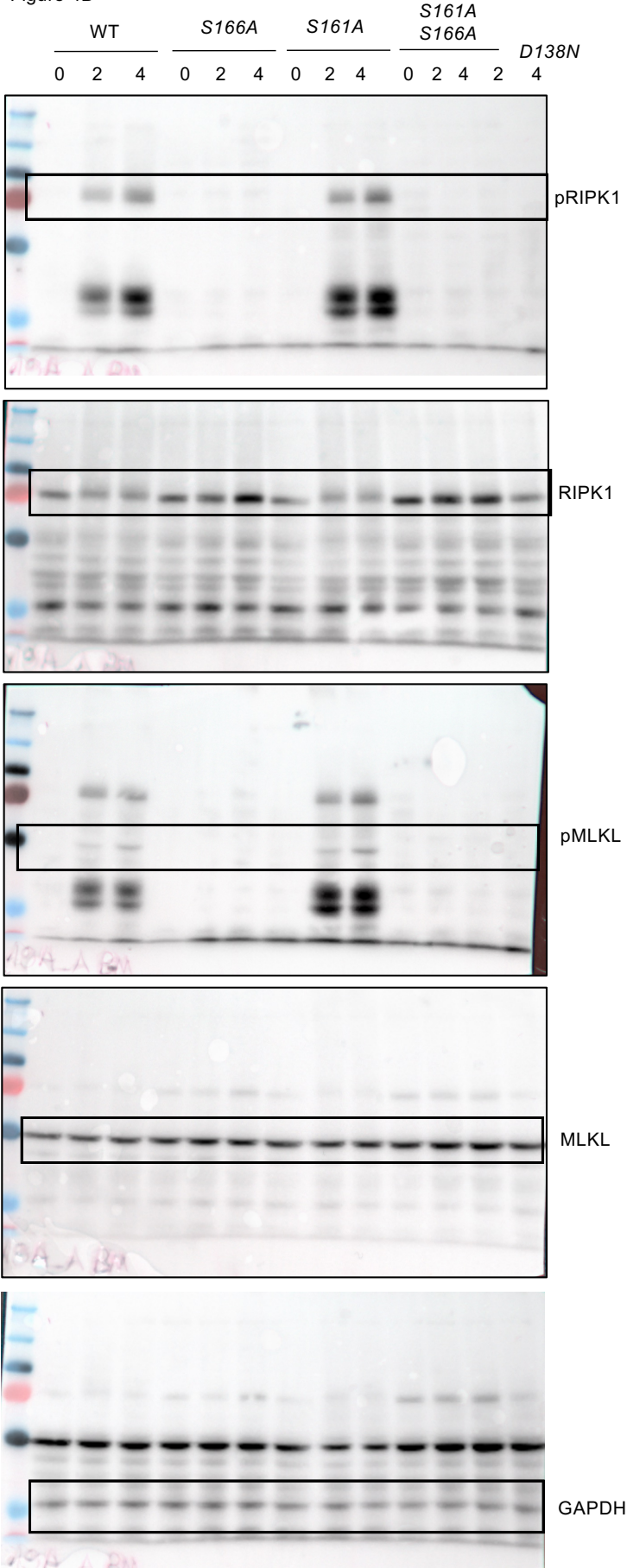

Figure 4C

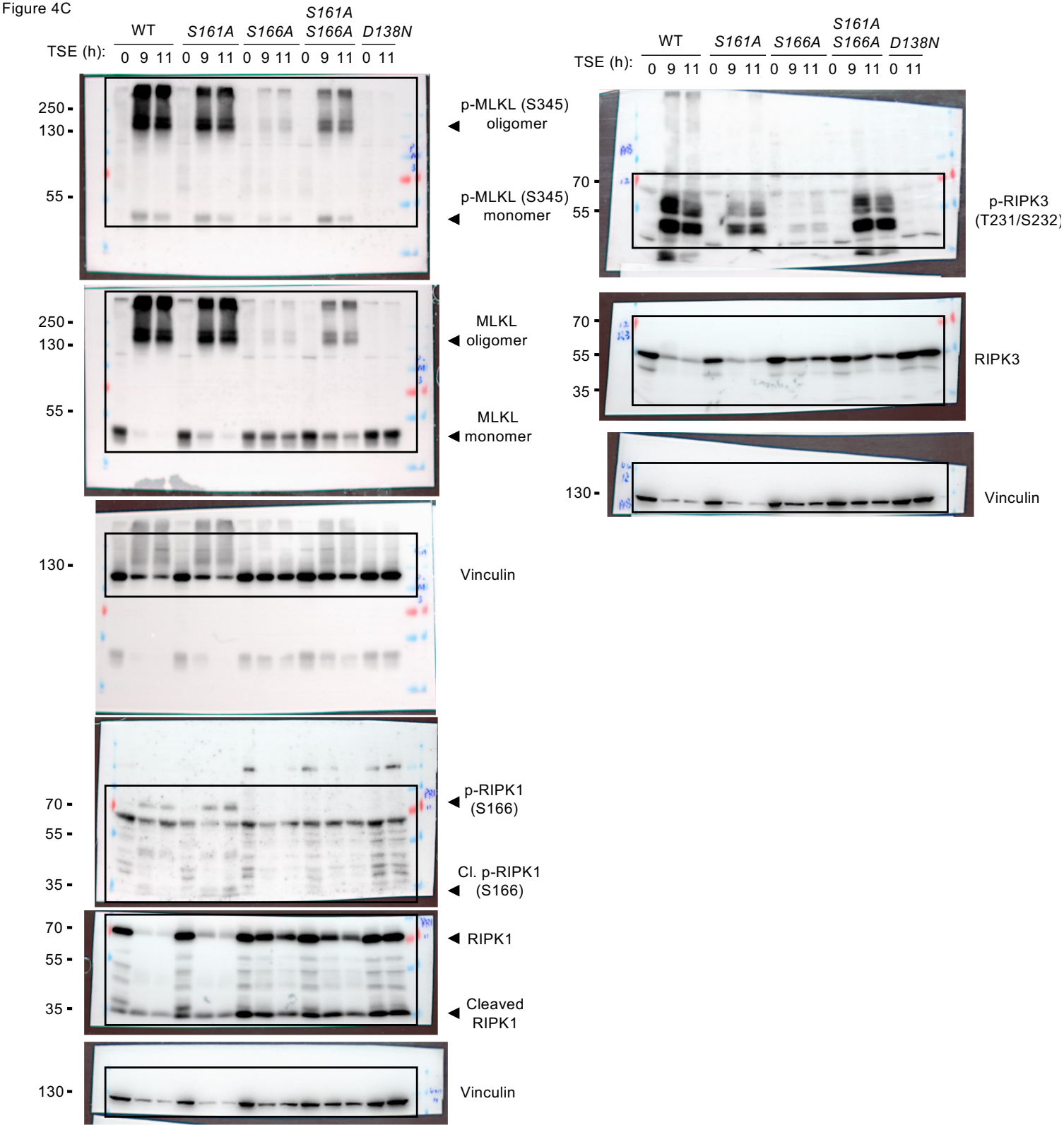

Figure 4E

|            | WT                                                                              |   |   |   |   | <i>S161A</i> |   |   |   |   | <i>S166A</i> |   |   |   |   | <i>S166A S161A</i> |   |   |   |   | <i>D138N</i> |  |  |  |  |
|------------|---------------------------------------------------------------------------------|---|---|---|---|--------------|---|---|---|---|--------------|---|---|---|---|--------------------|---|---|---|---|--------------|--|--|--|--|
| TAK1i (h): | 0                                                                               | 5 | 6 | 0 | 5 | 6            | 0 | 5 | 6 | 0 | 5            | 6 | 0 | 5 | 6 | 0                  | 5 | 6 | 0 | 5 | 6            |  |  |  |  |
| 1          | 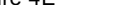  |   |   |   |   |              |   |   |   |   |              |   |   |   |   |                    |   |   |   |   |              |  |  |  |  |
| 2          | 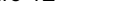  |   |   |   |   |              |   |   |   |   |              |   |   |   |   |                    |   |   |   |   |              |  |  |  |  |
| 3          | 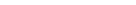  |   |   |   |   |              |   |   |   |   |              |   |   |   |   |                    |   |   |   |   |              |  |  |  |  |
| 4          | 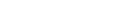  |   |   |   |   |              |   |   |   |   |              |   |   |   |   |                    |   |   |   |   |              |  |  |  |  |
| 5          | 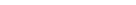  |   |   |   |   |              |   |   |   |   |              |   |   |   |   |                    |   |   |   |   |              |  |  |  |  |
| 6          | 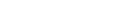  |   |   |   |   |              |   |   |   |   |              |   |   |   |   |                    |   |   |   |   |              |  |  |  |  |
| 7          | 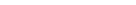  |   |   |   |   |              |   |   |   |   |              |   |   |   |   |                    |   |   |   |   |              |  |  |  |  |
| 8          | 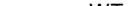  |   |   |   |   |              |   |   |   |   |              |   |   |   |   |                    |   |   |   |   |              |  |  |  |  |
| 9          | 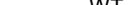  |   |   |   |   |              |   |   |   |   |              |   |   |   |   |                    |   |   |   |   |              |  |  |  |  |
| 10         | 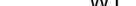 |   |   |   |   |              |   |   |   |   |              |   |   |   |   |                    |   |   |   |   |              |  |  |  |  |

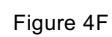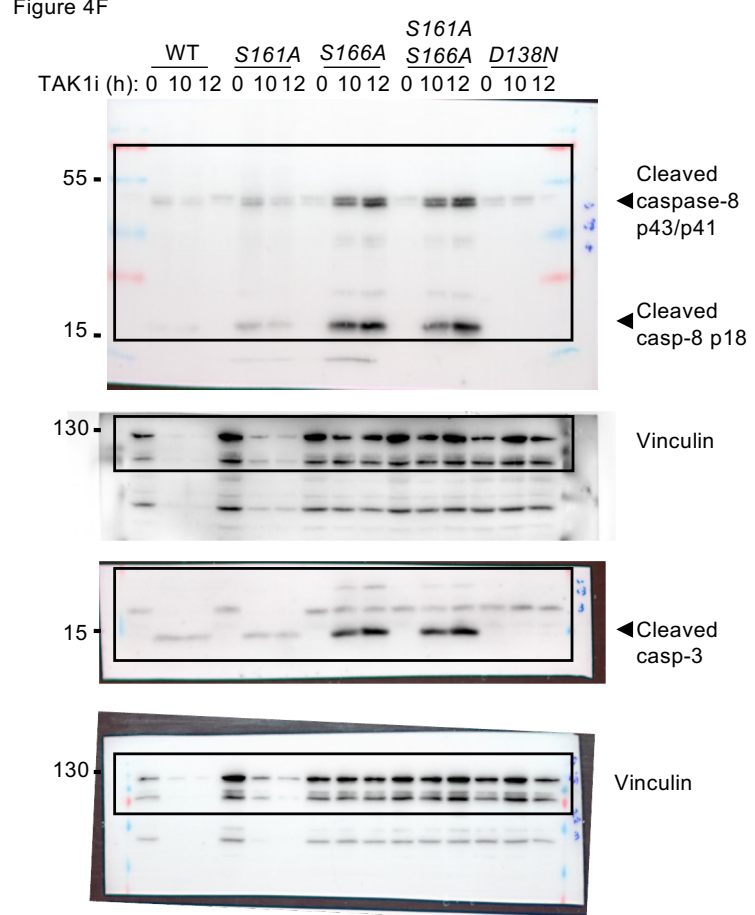

Supplement: SourceData F4 — is the source file for Fig. 4. [file jem_20250279_sourcedataf4.pdf]

Figure 5B

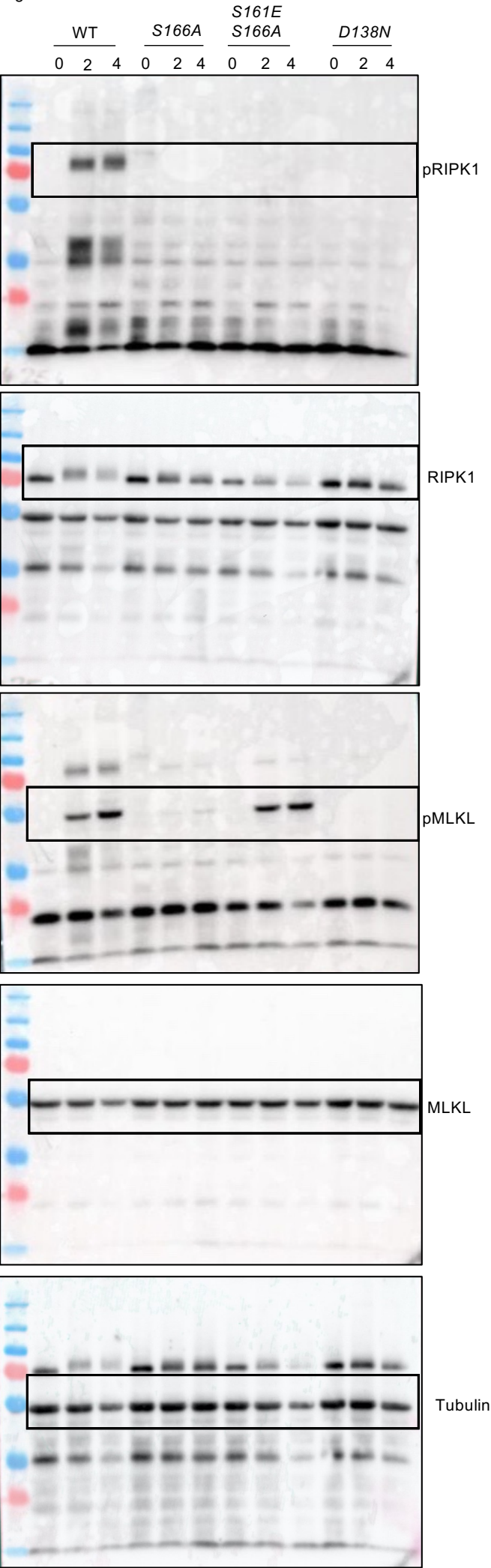

Supplement: SourceData F5 — is the source file for Fig. 5. [file jem_20250279_sourcedataf5.pdf]

Figure 6B

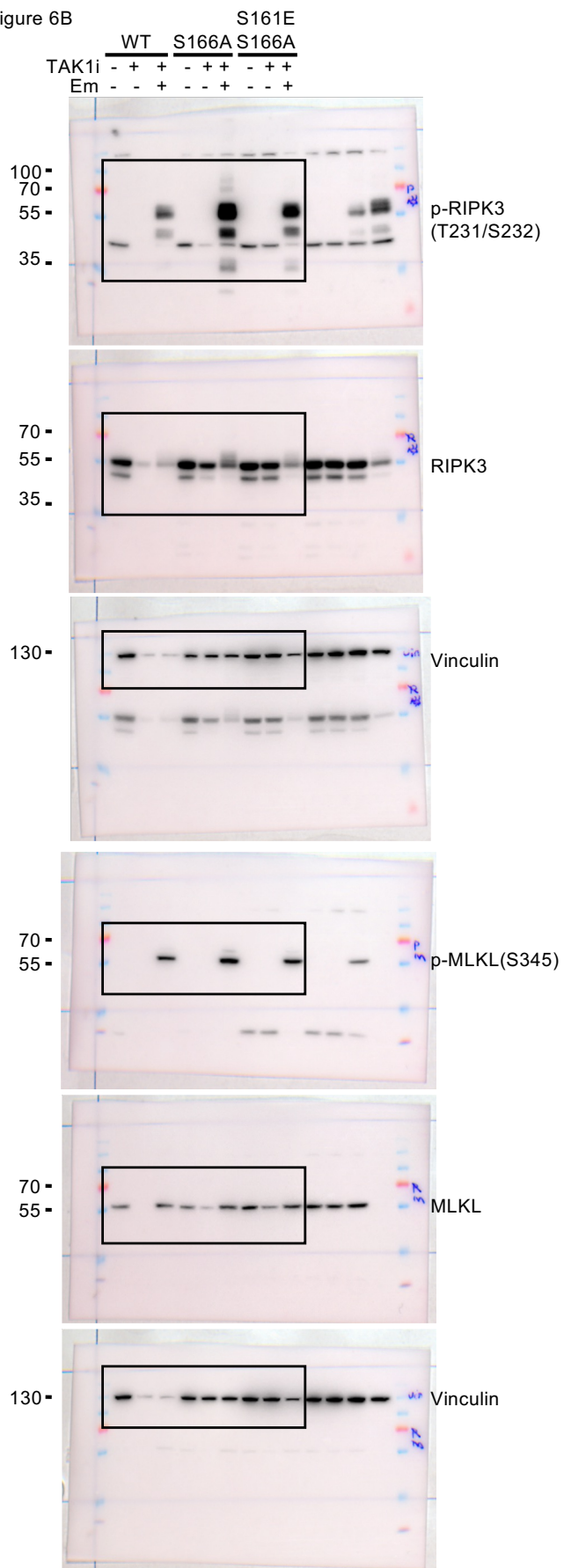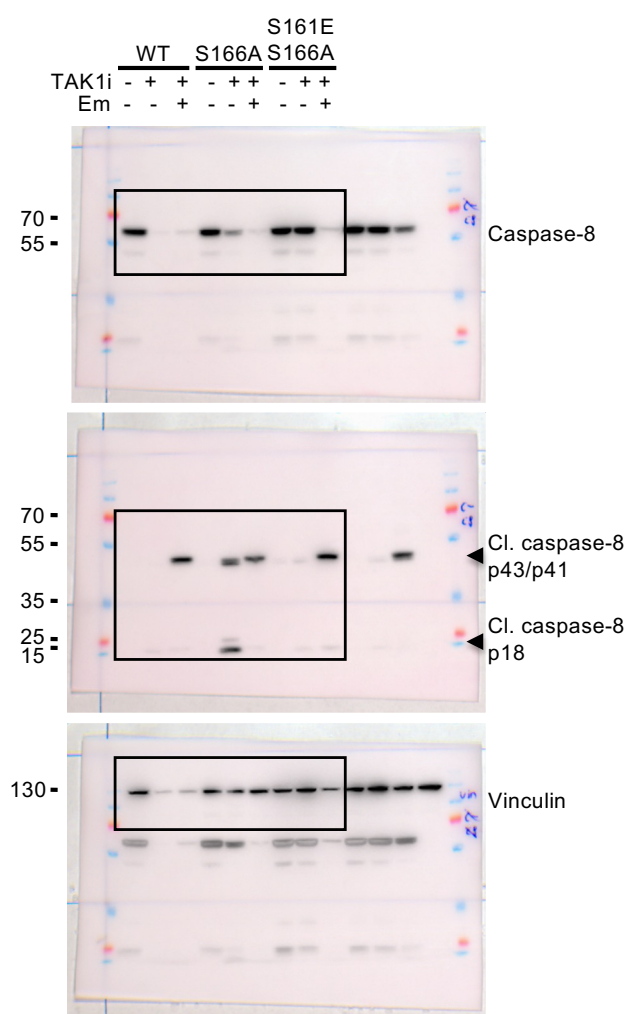

Supplement: SourceData F6 — is the source file for Fig. 6. [file jem_20250279_sourcedataf6.pdf]

Figure 7B

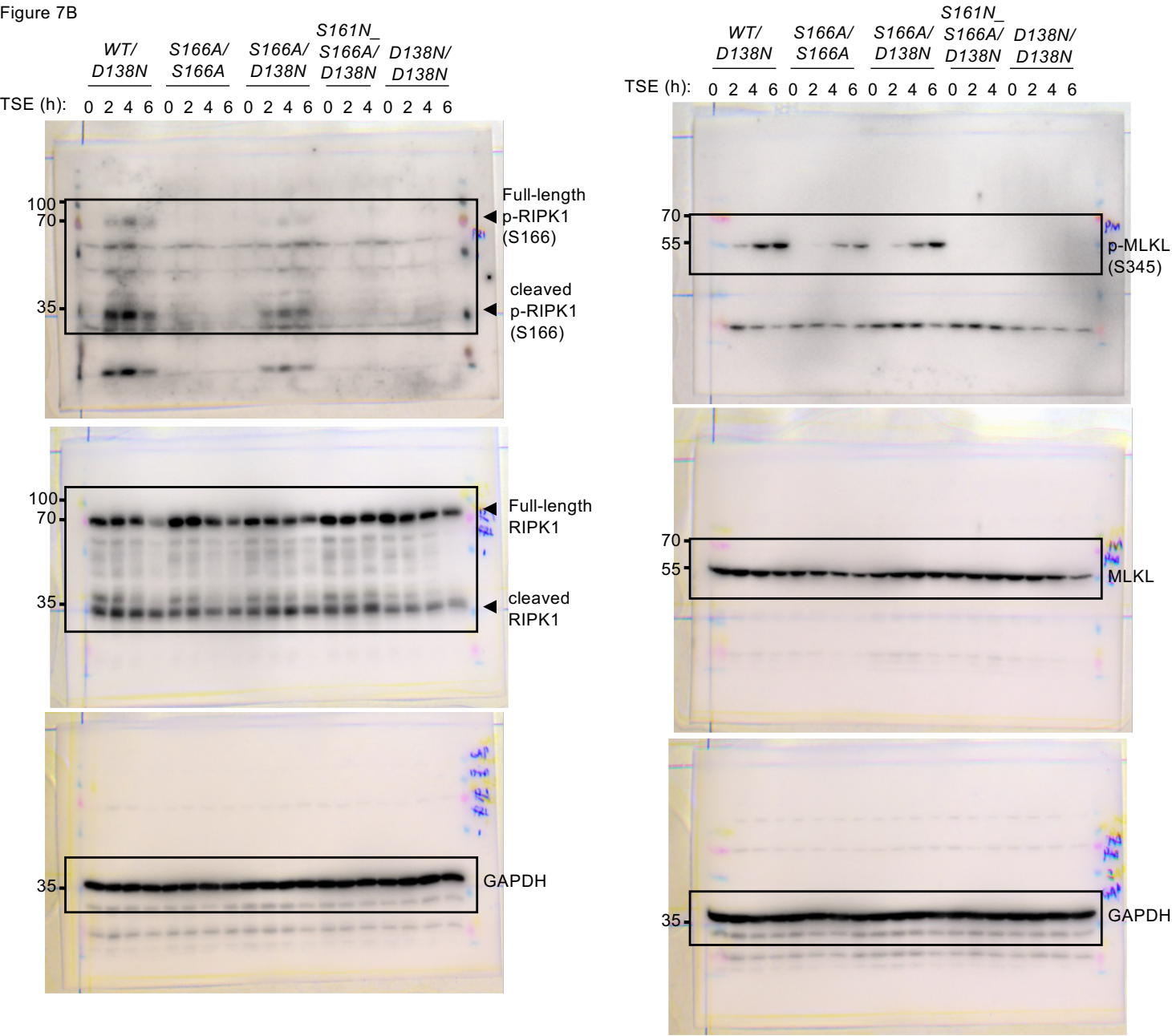

Figure 7C

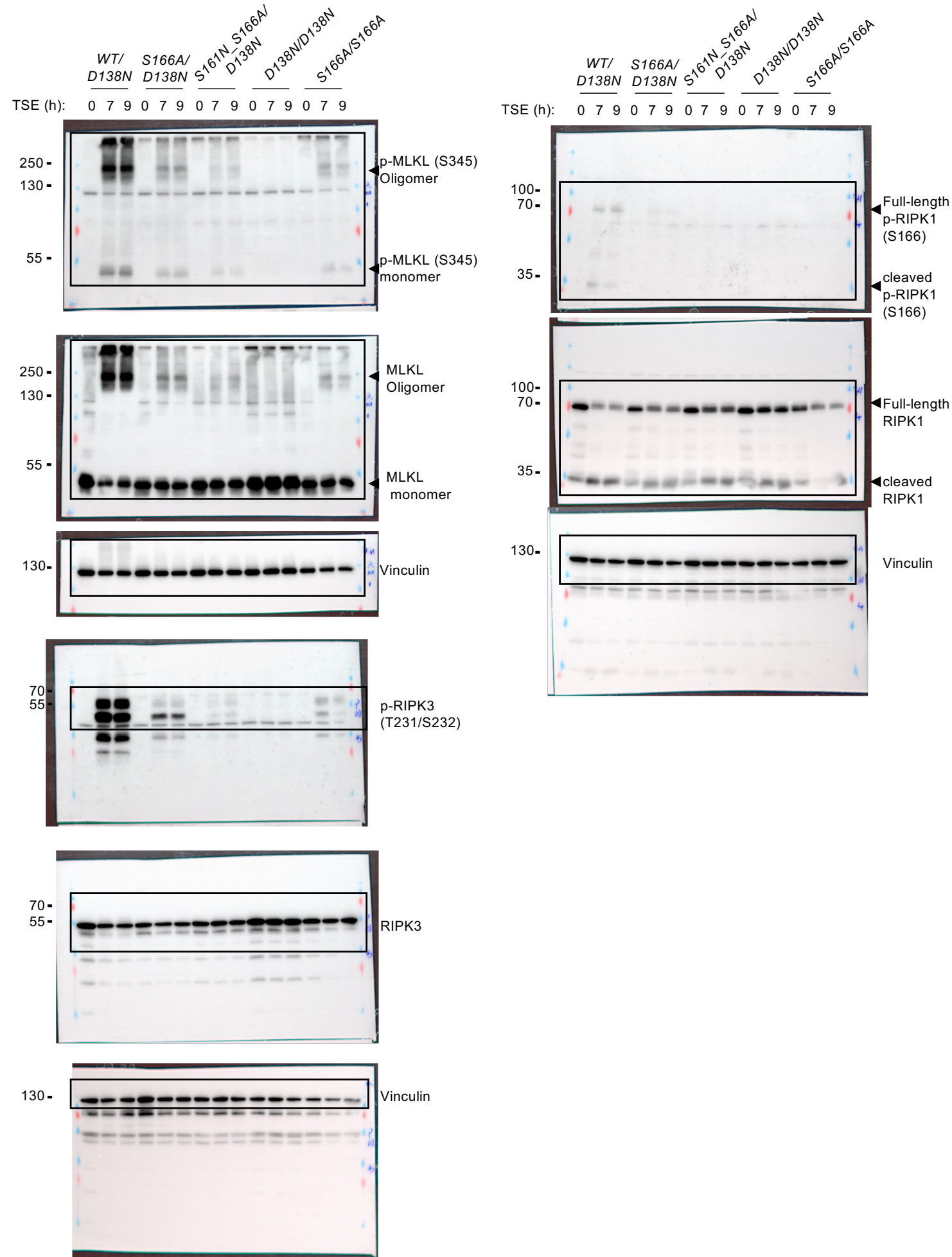

Figure 7E

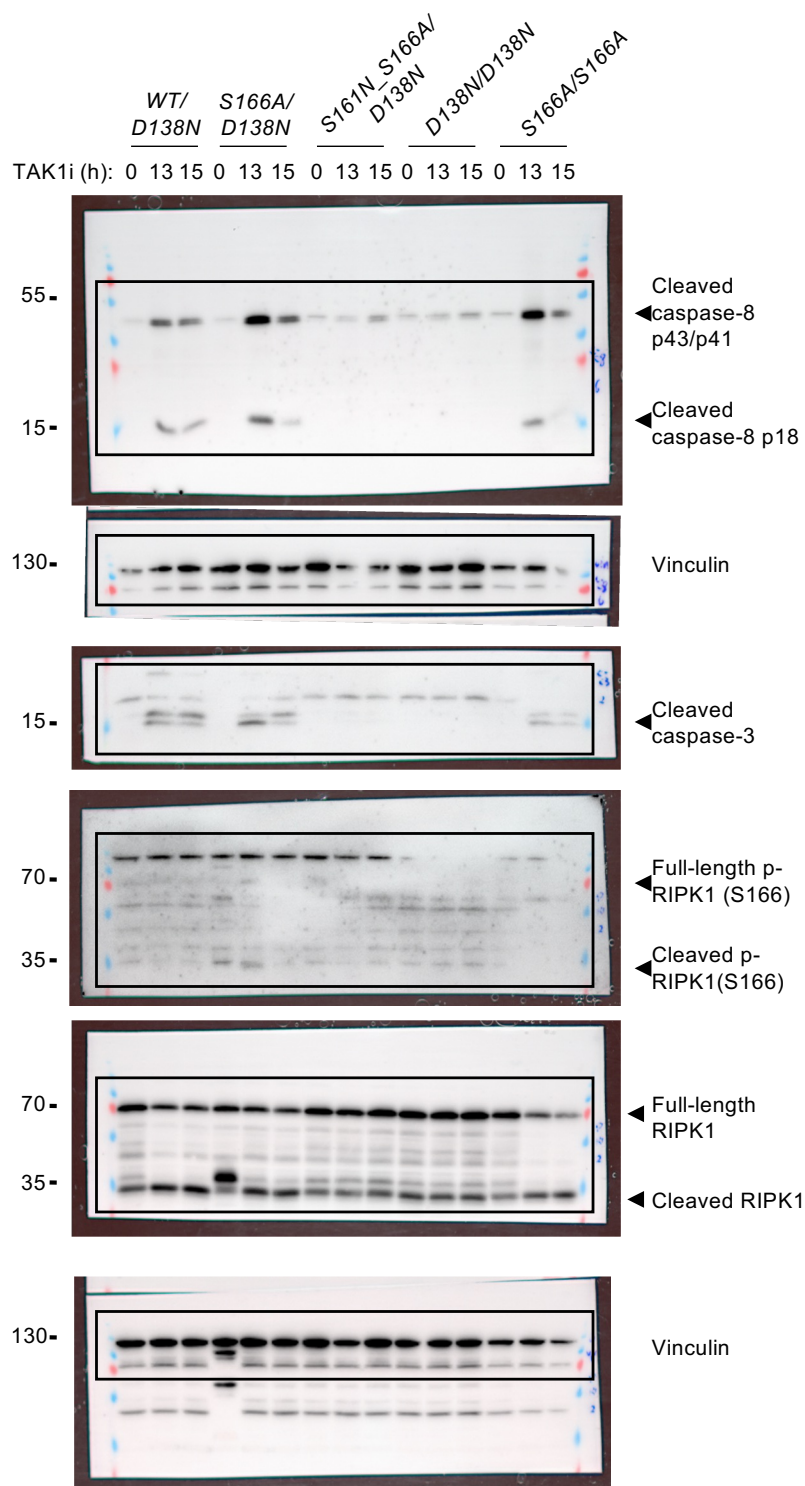

Supplement: SourceData F7 — is the source file for Fig. 7. [file jem_20250279_sourcedataf7.pdf]

Figure S2C

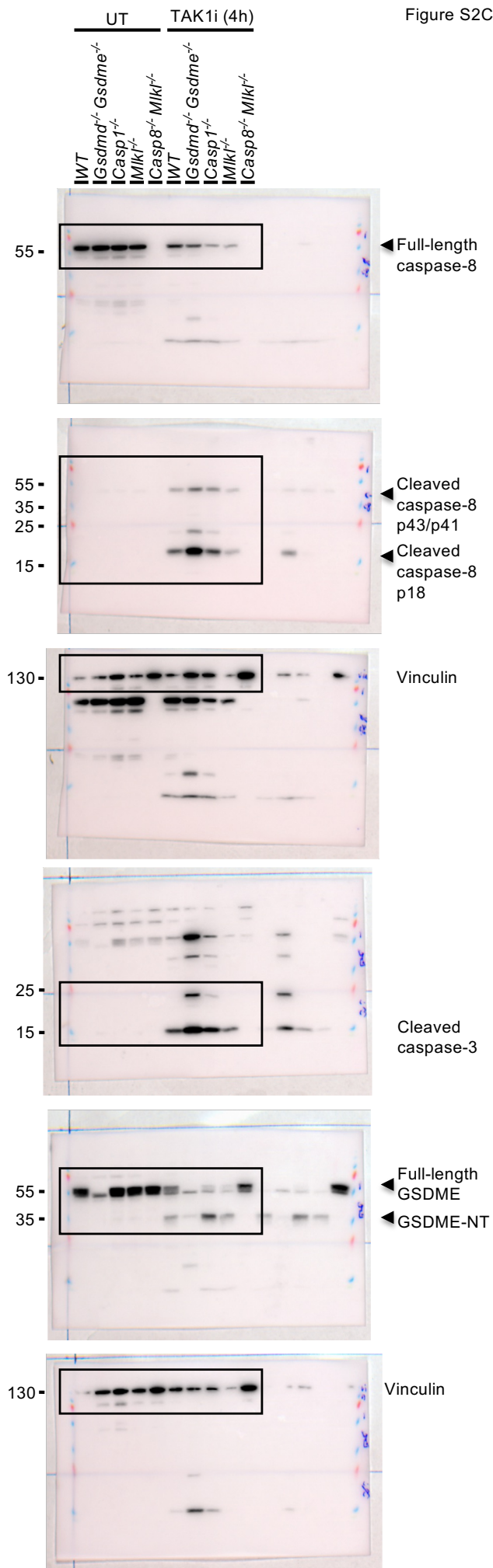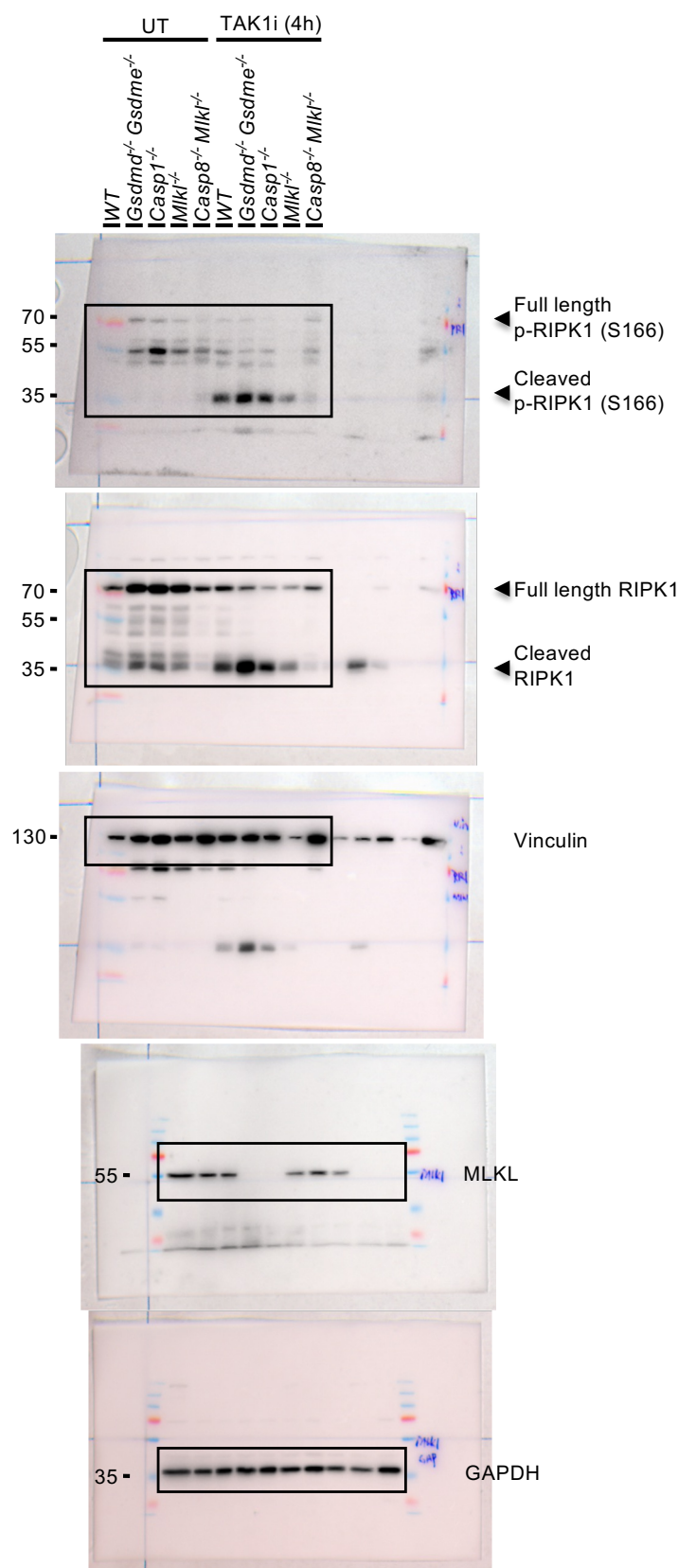

Figure S2C (continued)

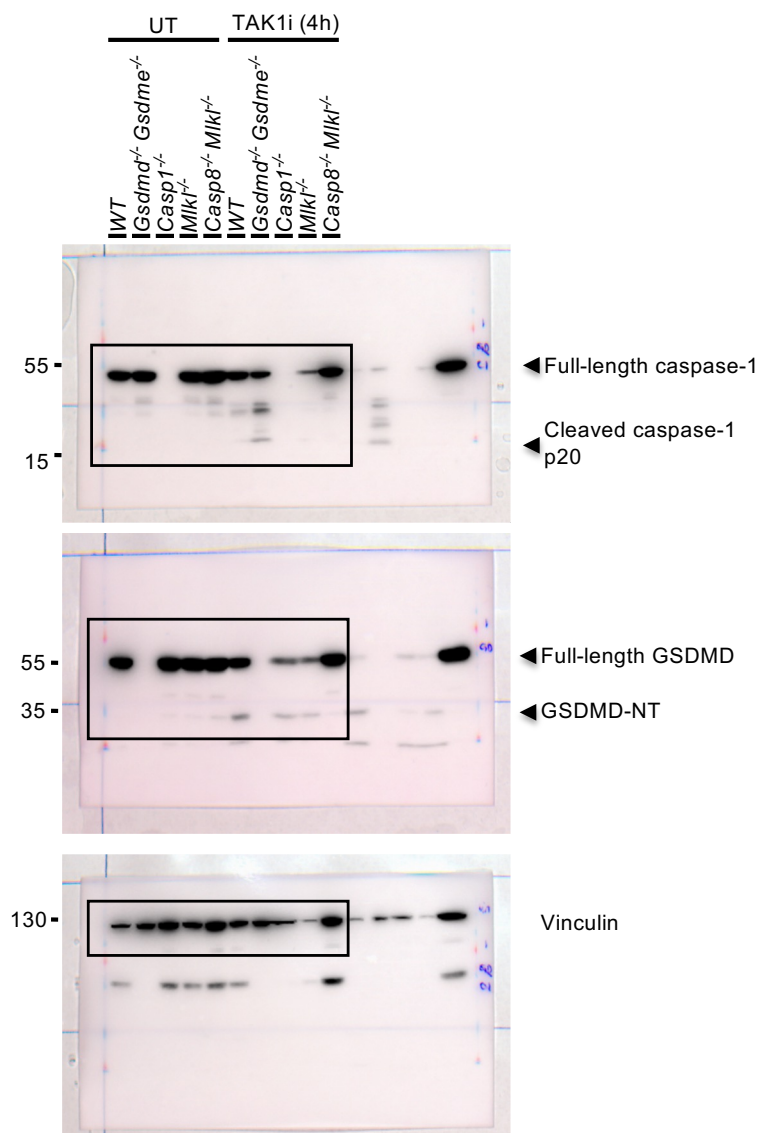

Supplement: SourceData FS2 — is the source file for Fig. S2. [file jem_20250279_sourcedatafs2.pdf]
